# Novel roles for LIX1L in promoting cancer cell proliferation through ROS1-mediated LIX1L phosphorylation

**DOI:** 10.1038/srep13474

**Published:** 2015-08-27

**Authors:** Satoki Nakamura, Tomoaki Kahyo, Hong Tao, Kiyoshi Shibata, Nobuya Kurabe, Hidetaka Yamada, Kazuya Shinmura, Kazunori Ohnishi, Haruhiko Sugimura

**Affiliations:** 1 Department of Tumor Pathology, 1-20-1 Handayama, Higashi-ku, Hamamatsu, Shizuoka 431-3192, Japan; 2 Equipment Center, 1-20-1 Handayama, Higashi-ku, Hamamatsu, Shizuoka 431-3192, Japan; 3 Cancer Center, Hamamatsu University School of Medicine, 1-20-1 Handayama, Higashi-ku, Hamamatsu, Shizuoka 431-3192, Japan

## Abstract

Herein, we report the characterization of Limb expression 1-like, (LIX1L), a putative RNA-binding protein (RBP) containing a double-stranded RNA binding motif, which is highly expressed in various cancer tissues. Analysis of MALDI-TOF/TOF mass spectrometry and RNA immunoprecipitation-sequencing of interacting proteins and the microRNAs (miRNAs) bound to LIX1L revealed that LIX1L interacts with proteins (RIOK1, nucleolin and PABPC4) and miRNAs (has-miRNA-520a-5p, −300, −216b, −326, −190a, −548b-3p, −7–5p and −1296) in HEK-293 cells. Moreover, the reduction of phosphorylated Tyr^136^ (pTyr^136^) in LIX1L through the homeodomain peptide, PY136, inhibited LIX1L-induced cell proliferation *in vitro*, and PY136 inhibited MKN45 cell proliferation *in vivo*. We also determined the miRNA-targeted genes and showed that was apoptosis induced through the reduction of pTyr^136^. Moreover, ROS1, HCK, ABL1, ABL2, JAK3, LCK and TYR03 were identified as candidate kinases responsible for the phosphorylation of Tyr^136^ of LIX1L. These data provide novel insights into the biological significance of LIX1L, suggesting that this protein might be an RBP, with implications for therapeutic approaches for targeting LIX1L in LIX1L-expressing cancer cells.

Non-specific cytotoxic therapies, such as chemotherapy and radiation, remain the cornerstone of therapy for patients with advanced cancer. To improve the effectiveness of anti-cancer therapies, it is necessary to identify molecular targets essential to tumor cells but dispensable for normal cells. In recent years, great strides have been made in understanding the molecular abnormalities that occur in tumor cells. The knowledge of the molecular pathogenesis of cancer has suggested new approaches for developing targeted therapies, and numerous molecular-targeted drugs have been introduced for the treatment of advanced malignancies.

To identify differences in the transcriptional responses between acute myeloblastic leukemia (AML) cells and normal hematopoietic progenitor cells, transcriptional alterations have been investigated using microarrays encompassing the entire human genome. Approximately 180 genes belonging to functional categories, such as transcription factors, regulators of the cell cycle and cell proliferation and proteins involved in signaling pathways, have shown increased expression at the transcriptional level in AML cells^1^. Among these genes, *Limb expression 1-like* (*LIX1L*) was identified as a key regulator of cell proliferation. *Limb expression 1 (LIX1*), the vertebrate homolog of *lowfat* (*Lft*), and *LIX1L* were originally isolated as genes expressed in chicken limbs^2^. *LIX1* was first identified in chickens through a differential screen for genes expressed during early limb development^2^. A subsequent analysis in mice revealed that *LIX1* has a substantially broader expression pattern^3^. *LIX1L* has only been defined based on sequence similarity to *LIX1* and contains a double-stranded RNA binding motif and is likely an RNA-binding protein (RBP). RBPs have been demonstrated as key regulators of gene expression^4^,^5^. However, the biological functions of the *LIX1L* gene, located on chromosome 1q21.1, have not been described, and little is known regarding the expression and role of this protein in cancer cells.

Homeodomain (also called mimetic) peptides for the activated domains of oncogenic genes can inhibit or neutralize gene function^6^,^7^,^8^,^9^. Because LIX1L promotes cancer cell proliferation, in the present study, we investigated the expression and function of LIX1L *in vitro* and examined the effects of this protein on *in vivo* tumor growth. We found that the gene and protein expression of LIX1L is increased in esophageal, gastric, breast, lung, thyroid, ovarian, kidney, liver, colon, prostate and pancreatic cancer cells. Moreover, we identified LIX1L-targeting tyrosine kinases and LIX1-mediated miRNA expression, showing that LIX1L PY136 induced tumor cell apoptosis.

## Results

### LIX1L expression in human tumor samples as detected through IHC and western blot analyses

**Figure 1:**
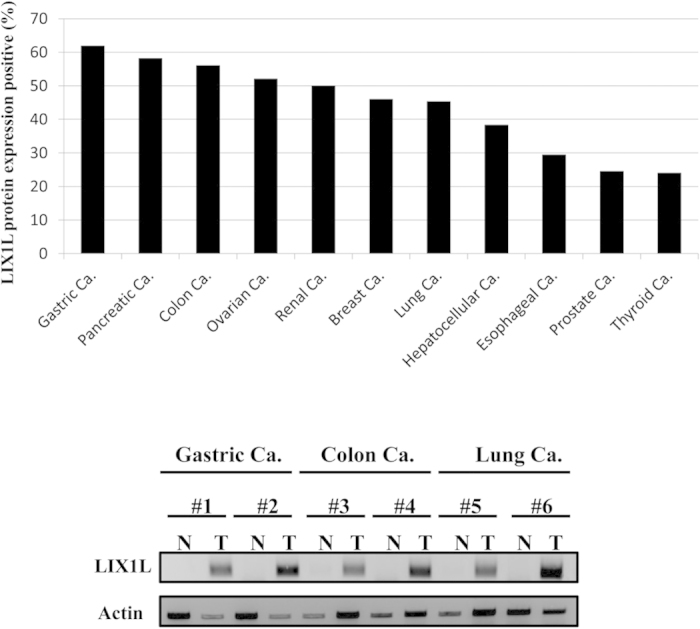
Immunohistochemical (IHC) staining for LIX1L in cancer tissues. IHC staining of human solid tumor tissue. Gastric cancer (n = 540), pancreatic cancer (n = 43), colon cancer (n = 50), ovarian cancer (n = 50), renal cancer (n = 58), breast cancer (n = 50), lung cancer (n = 64), hepatocellular cancer (n = 47), esophageal cancer (n = 51), prostate cancer (n = 53) and thyroid cancer (n = 50) samples were evaluated (upper panel). A score of 2 or 3 indicated positive LIX1L expression. LIX1L protein expression levels in the frozen surgical specimens (gastric cancer, #1 and #2; colon cancer, #3 and #4; lung cancer, #5 and #6) were assessed using western blotting. Actin was immunoblotted as a loading control. Western blotting results representing three independent experiments are shown (bottom panels). N, normal tissues; T, tumor tissues.

As shown in Fig. 1, LIX1L was strongly expressed in 61.9% of gastric cancer samples (n = 540), 58.1% of pancreatic cancer samples (n = 43), 56% of colon cancer samples (n = 50), 52% of ovarian cancer samples (n = 50), 50% of renal cancer samples (n = 58), 46% of breast cancer samples (n = 50), 45.3% of lung cancer samples (n = 64), 38.3% of hepatocellular cancer samples (n = 47), 29.4% of esophageal cancer samples (n = 51), 24.5% of prostate cancer samples (n = 53) and 24% of thyroid cancer samples (n = 50) (upper panel). LIX1L was confirmed to be overexpressed in protein extracts from frozen surgical specimens (gastric, colon, and lung cancer). LIX1L was also more strongly expressed in tumor tissues than in normal tissues (bottom panels). Representative photomicrographs are provided in Supplementary Figure 1. The subcellular localization was predominantly cytoplasmic.

The following normal tissues showed negative staining for LIX1L expression, represented as a staining score of 0 or 1: esophagus, stomach, colon, thyroid, liver, prostate, breast, lung and ovary (Supplementary Figure 2). Moreover, normal brain tissues showed weak LIX1L expression. Normal cardiac muscle also showed no LIX1L expression (data not shown).

### Effects of LIX1L knockdown on gastric cancer cell proliferation

**Figure 2:**
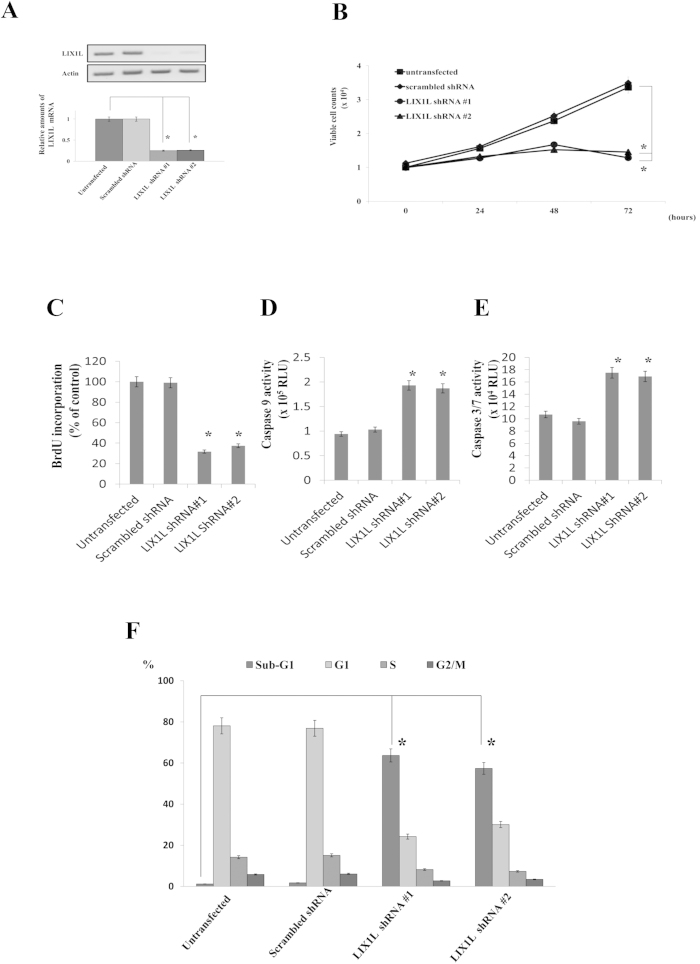
The effects of *LIX1L* knock-down on gastric cancer cell proliferation. OCUM-1 cells were transfected with scrambled shRNA or *LIX1L* shRNA-#1 or -#2. (**A**) LIX1L protein expression levels in cells were assessed using western blotting. Actin was included as a loading control. Western blotting results representing three independent experiments are shown (upper panels). The *LIX1L* mRNA level was analyzed using quantitative RT-PCR, and the level was determined relative to that of *GAPDH* (bottom panel). (**B**) At three days post-transfection, the number of viable cells was counted every 24 h for three days. *p < 0.01 compared with untransfected control cells. (**C**) BrdU incorporation was determined through ELISA at 24 h. (**D**,**E**) The activities of caspases-3/7 and −9 were determined 48 h after transfection. (**F**) The cell cycle distribution of OCUM-1 cells was analyzed using flow cytometric analysis at 72 h after transfection. The fractions of cells in the G1, S and G2/M stages of the cell cycle were determined. The results are presented as the mean ± SD from three independent experiments. *p < 0.01.

To examine the functional importance of LIX1L expression in cancer cells, we first examined the effects of LIX1L knockdown on gastric cancer cell proliferation. OCUM-1 gastric cancer cells were transfected with *LIX1L* shRNA-#1 or -#2 (Fig. 2A and Supplementary Figure 3), and the effects of the LIX1L knockdown on OCUM-1 proliferation were assessed over 72 h of culture, starting from day 3 post-transfection. The results showed that *LIX1L* shRNA-#1 and -#2 mediated *LIX1L* mRNA expression knockdown by 75% and 74%, respectively. Cell proliferation was measured by counting the cells using trypan blue exclusion (Fig. 2B). When the OCUM-1 cells were transfected with *LIX1L* shRNA-#1 or -#2, cell proliferation was significantly decreased compared with untreated cells and cells transfected with scrambled shRNA. Moreover, *LIX1L* knockdown in other gastric cancer cell lines (KATO-III and MKN45) similarly reduced proliferation (data not shown).

Next, the incorporation of BrdU was measured, and the rate of DNA synthesis at 24 h was determined after transfection in OCUM-1 cells, showing that BrdU incorporation was significantly reduced after LIX1L knockdown (Fig. 2C). Moreover, to determine whether the growth inhibition induced through LIX1L knockdown was associated with apoptosis, the caspase activities in OCUM-1 cells were determined at 48 h after transfection with *LIX1L* shRNA #1 or #2. The results showed that the activities of caspases-3/7 and −9 were significantly increased in LIX1L-knockdown OCUM-1 cells (Fig. 2D,E) compared with control cells, suggesting that OCUM-1 growth suppression through LIX1L knockdown might reflect the induction of apoptosis. Similarly, in other gastric cancer cell lines (KATO-III and MKN45), LIX1L knockdown also suppressed BrdU incorporation and induced caspase-3/7 and −9 activities (data not shown).

We further examined the effects of LIX1L knockdown on gastric cancer cell proliferation through a flow cytometric analysis of the effects of LIX1L on cell cycle distribution. As shown in Fig. 2F, the analysis of OCUM-1 cells on day 3 post-transfection with *LIX1L* shRNA-#1 or -#2 indicated an increase in the percentage of cells in the sub-G1 phase compared with the cells treated with scrambled shRNA or vehicle alone. No increase in the polyploid (>4N) population of these cells was observed.

LIX1L knockdown also induced apoptosis in other gastric cancer cell lines (KATO-III and MKN45) according to a caspase activity assay (data not shown). These results showed that a reduction of LIX1L expression strongly inhibited gastric cancer cell proliferation and induced apoptosis in these cells.

### The *in vitro* and *in vivo* anti-cancer activity of the homeodomain peptide, PY136

**Figure 3:**
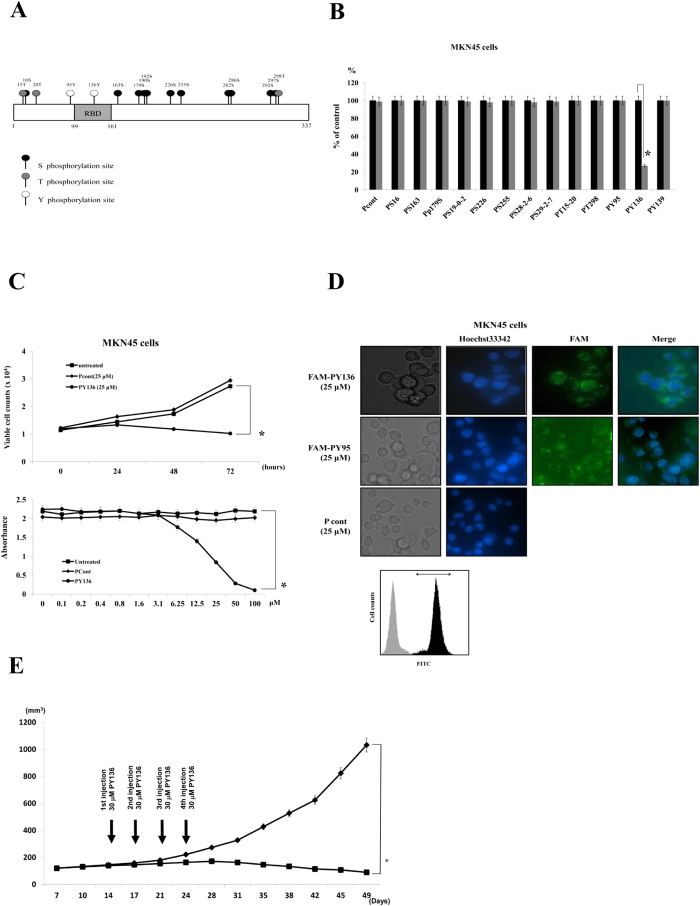
A LIX1L homeodomain peptide inhibits tumor growth. (**A**) A schematic diagram showing the domain structure of the human LIX1L protein containing a RBD, predicted serine phosphorylation sites, predicted threonine phosphorylation sites and predicted tyrosine phosphorylation sites. (**B**) The cell viability with viable cell counts. MKN45 cells were treated with the control peptide or one of 13 homeodomain peptides (50 μM) for 72 h. (**C**) The cell proliferation was assessed based on the viable cell counts (upper panel) and MTT assay (bottom panel) in cells treated with the indicated concentrations of peptides for 72 h. MKN45 cells were treated with Pcont (25 μM) and PY136 (25 μM). *p < 0.001 (**D**) Confirmation of 25 μM FAM-PY136 and 25 μM FAM-PY95 internalization (green) into non-permeabilized MKN45 cells after a three-hour treatment. The nuclei were stained using Hoechst 33342 (blue). The cells were viewed using phase-contrast and fluorescence microscopy, and 25 μM Pcont was used negative control. Representative images are shown (magnification, 400x) (upper panels). MKN45 cells treated with FAM-PY136 were analyzed using flow cytometry. Negative control MKN45 cells; light gray region, FAM-PY136-treated MKN45 cells; dark gray region (bottom panel). The arrow indicates the FITC-positive region. (**E**) Tumor treatment with PY136 (30 μM; n = 3). Cohorts of nude mice bearing size-matched human gastric cancer xenografts (MKN45-derived) were used for the *in vivo* studies. *p* < 0.05.

Next, we examined the inhibition of LIX1L function in cancer cells. As shown in Fig. 3A, LIX1L encodes one double-stranded RNA-binding domain (dsRBD), but the function of the other domain remains unclear. Previous analyses have shown that the function of RBPs (TIS11b and KSRP) is controlled and coordinated through phosphorylation^10^. Thus, RBPs might represent a point of convergence for the activity of different kinases, such as phosphoinositide-3-kinase (PI3K) and mitogen-activated protein kinase (MAPK), which regulate RBP localization and function^11^. Upon activation, PKB and MK2 phosphorylate TIS11b at Ser^92^ and Ser^203^, and subsequently, TIS11b is prevented from mediating ARE-mediated mRNA decay^12^,^13^. Similarly, but not at same phosphorylation sites, KSRP is phosphorylated at Ser^193^ and Thr^692^ by PKB and p38 respectively^14^,^15^. LIX1L has 11 serine, three threonine and two tyrosine residues predicted to undergo phosphorylation. Therefore, we hypothesized that the phosphorylation of LIX1L would play an important role in the activities of this protein in cancer cells. To examine this hypothesis, we synthesized various homeodomain peptides containing 10 amino acid residues corresponding to the areas of LIX1L with predicted phosphorylation sites (Ser, Thr and Tyr residues; Table 1). These peptides might inhibit LIX1L phosphorylation as decoys. Surprisingly, only one homeodomain peptide, PY136, inhibited the proliferation of MKN45 cells (Fig. 3B,C). None of the other homeodomain peptides affected MKN45 cell proliferation. Similar effects of PY136 were observed in OCUM-1 and KATOIII cells. However, PY136 did not inhibit the proliferation of NUGC-4 cells, which do not express *LIX1L* mRNA (Supplementary Figure 4). We confirmed the internalization of PY136 in NUGC-4 cells (Supplementary Figure 5).

We subsequently investigated whether a random homeodomain peptide would inhibit cancer cell proliferation (Supplementary Figure 6). Similarly, only PY136 inhibited the proliferation of KATO-III and OCUM-1 cells. We also investigated whether the inhibitory effects of PY136 were dependent on peptide length. The results showed that the inhibitory effects of PY136 were independent of the length of the peptide in KATO-III and OCUM-1 cells (using 10-, 20- and 30-aa peptides), and none of these peptides inhibited the proliferation of NUGC-4 cells (Supplementary Figure 7).

The internalization of the homeodomain peptide PY136 was examined after fusing this domain with a TAT sequence (YGRKKRRQRRR) and performing a receptor-independent cell entry assay. PY136, PY95 and Pcont internalization and cytoplasmic localization was observed in 92, 95 and 91% of MKN45 cells, respectively, using the flow cytometry at 3 h post-treatment (Fig. 3D). The uptake rates of the other peptides indicated in Fig. 3B was also approximately 90% in MKN45 cells. The inhibition of tumor growth through PY36 in tumor-bearing mice was also examined. As shown in Fig. 3E, marked inhibition of tumor growth was observed after the subcutaneous (s.c.) administration of PY136. In contrast, control peptides showed no effects on tumor growth. These findings suggest that the 10-aa sequence PSNSPPYVCY might be the functional motif responsible for the pro-proliferative activity of LIX1L.

### Oncogenic activity of LIX1L and identification of proteins associated with LIX1L using proteome analysis

**Figure 4:**
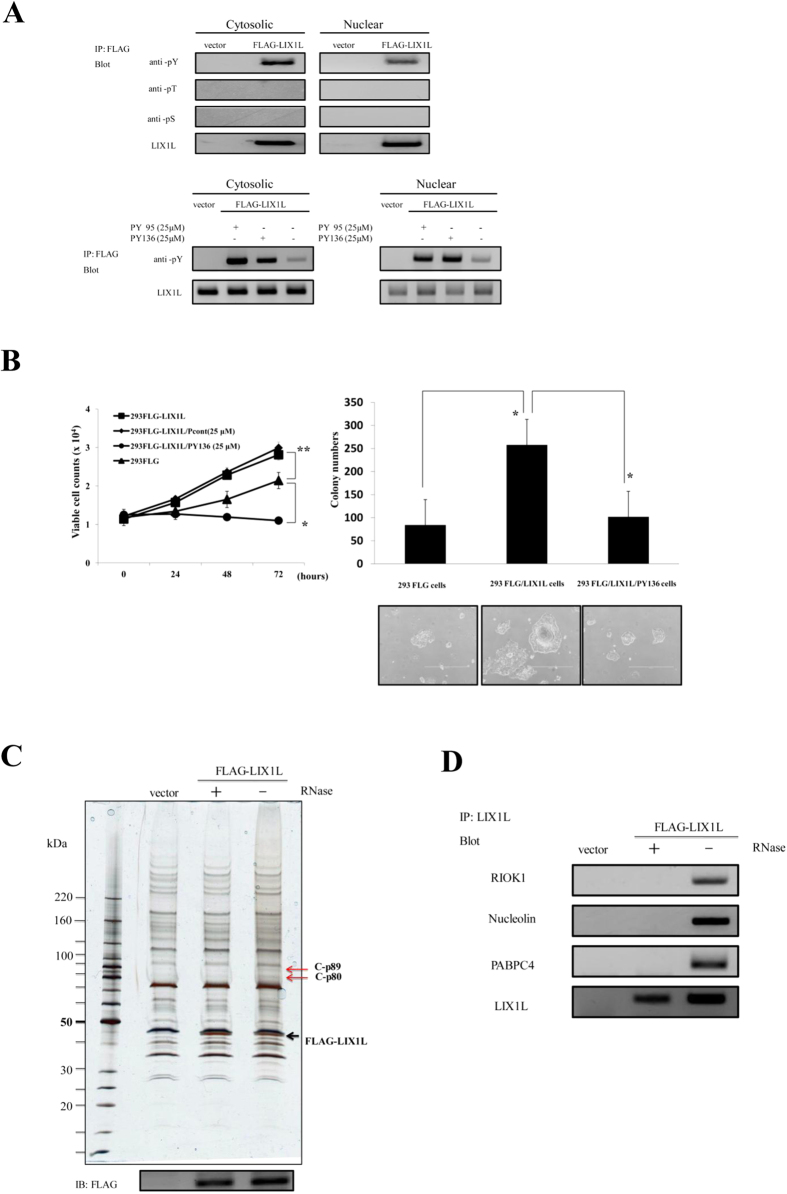
LIX1L-mediated oncogenic effects and identification of LIX1L-associated proteins using a proteome analysis. (**A**) Phosphorylated LIX1L was immunoprecipitated from the cytosolic and nuclear fractions of HEK-293FLG and HEK-293FLG-LIX1L cells using a FLAG antibody. Immunoprecipitates were analyzed through western blot analysis with a LIX1L antibody and phosphorylated serine-, threonine- and tyrosine-specific antibodies (upper panels). In the cytosolic and nuclear fractions of the HEK-293FLG and HEK-293FLG-LIX1L cells treated with 25 μM PY95 as a negative control or PY136, immunoprecipitates obtained using the FLAG antibody were analyzed through a western blot analysis with the LIX1L antibody and phosphorylated tyrosine-specific antibodies (bottom panels). Representative blots from HEK-293FLG and HEK-293FLG-LIX1L cell lines are shown. (**B**) The cell counts of HEK-293FLG and HEK-293FLG-LIX1L cells after treatment with PY136 (left panel). The HEK-293FLG and HEK-293FLG-LIX1L cells were cultured in semisolid methylcellulose media. The HEK-293FLG-LIX1L cells were left untreated or were treated with PY136 (25 μM). After 14 days in culture, colony formation was analyzed, and the cells were viewed using phase-contrast microscopy. The colonies formed from each cell type (3 × 10^2^ to 5 × 10^2^ cells/plate) were counted following plating onto semisolid methylcellulose media (right upper panel). Original magnification 4x (right bottom panels). These data are shown as the mean ± SD for independent experiments. **p < 0.05, *p *<* *0.01*. (**C**) The results of the immunoblot analysis of the cytosolic fraction treated with or without RNase in HEK-293FLG-LIX1L cells. The black arrow indicates the FLAG-LIX1L fusion protein. The red arrows indicate the detected proteins associated with the LIX1L-RNA complex. (**D**) Western blot analysis revealed that LIX1L interacted with the RIOK1, nucleolin and PABPC4 proteins in the cytoplasm of HEK-293 cells. In (**A**) and (**D**), the cropped blots were run under the same experimental condition.

To investigate the biological function of LIX1L in cancer cells, we first assessed LIX1L phosphorylation in human embryo kidney (HEK)-293 (HEK-293) cells. As shown in Fig. 4A (upper panels), the phosphorylation of the tyrosine residues of LIX1L, but not of the serine and threonine residues, was detected in both the cytosolic and nuclear fractions of FLAG-LIX1L expressing HEK-293 (HEK-293FLG-LIX1L) cells. The activity of anti-pThr and pSer antibodies was confirmed, showing the positive controls of these antibodies (Supplementary Figure 8) and the accurate separation of cytosolic and nuclear fractions (Supplementary Figure 9). Moreover, PY136 inhibited the phosphorylation of tyrosine residues in both of these fractions, but these effects were not observed with PY95 as a negative control peptide (Fig. 4A, bottom panels). Next, the inhibitory effects of LIX1L on cell proliferation were assessed using cell counts and a colony-formation assay (Fig. 4B). The results showed that the numbers of HEK-293FLG-LIX1L cells were significantly increased compared with those of FLAG-expressing HEK-293 (HEK-293FLG) cells, and the viable cell counts of HEK-293FLG-LIX1L cells decreased after treatment with PY136 (Fig. 4B, left panel). In contrast, the other homeodomain peptides did not inhibit the proliferation of HEK-293FLG-LIX1L cells (Supplementary Figure 10). Moreover, LIX1L transfection also increased the numbers of HEK-293FLG-LIX1L cells, and pY136 inhibited HEK-293FLG-LIX1L colony formation in soft agar (Fig. 4B, right panels). These results suggest that LIX1L drives the proliferation of HEK-293 cells, and PY136 shows LIX1L expression-dependent inhibition of cell proliferation.

To determine whether the LIX1L protein is associated with other proteins involved in cancer cell proliferation, immunoprecipitation using an anti-FLAG antibody was performed in HEK-293FLG-LIX1L cells (Fig. 4C). The cells were separated into cytoplasmic and nuclear fractions, and the supernatants derived from each fraction were detected through silver staining. When the supernatant from the cytoplasmic fraction was treated with RNase, no differentially expressed proteins were detected in HEK-293FLG-LIX1L cells. In contrast, when the supernatant from the cytoplasmic fraction was treated without RNase, differentially expressed proteins were detected in these cells. These results indicate that the LIX1L protein is associated with other proteins, C-p89 and C-p80 kDa, through the formation of a complex with the RNA in the cytoplasmic fraction. Moreover, when the supernatant from the nuclear fraction was treated without RNase, differentially expressed proteins were detected in HEK-293FLG-LIX1L cells (Supplementary Figure 11). These differentially expressed proteins were identified through MALDI-TOF/TOF mass spectrometry (Table 2).

To ensure consistency in the observed differential expression, a western blot analysis was performed using the cytoplasmic fraction from HEK-293FLG-LIX1L cells. Among these proteins, high immunoreactivity was observed for RIOK1 (C-p89), nucleolin (C-p80) and PABPC4 (C-p80) (Fig. 4D). No other proteins were detected in the cytoplasmic fraction. In the nuclear fraction, the differentially expressed proteins identified through MALDI-TOF/TOF mass spectrometry (Supplementary Table 1) were DHX9 (N-p150), nucleolin (N-p100) and hnRNPL (N-p68) (Supplementary Figure 12). These results demonstrate that the LIX1L protein might form a complex comprising RIOK1, nucleolin and PABPC4 proteins with certain RNAs in the cytoplasm and DHX9, nucleolin and hnRNPL with certain RNAs in the nucleus of HEK-293FLG-LIX1L cells.

### Analysis of the LIX1L mediated-miRNA expression by next-generation sequencing

Next, we investigated and identified LIX1L-binding RNAs using an RNA immunoprecipitation (RIP) assay and next-generation sequencing (NGS). The total number of sequencing reads obtained was 43.5 and 47.4 million reads per sample from the HEK-293FLG and HEK-293FLG-LIX1L cells, respectively. After filtering, the sequencing reads with lengths between 18 and 29 nucleotides represented 14.4% (HEK-293FLG) and 16.3% (HEK-293FLG-LIX1L) of the total number of reads obtained initially. A total of 31.3% (HEK-293FLG) and 24.4% (HEK-293FLG-LIX1L) of these reads were mapped to the human genome. We detected 8,221 small RNAs (7,768 known and 453 novel RNAs) and 2,104 miRNAs. Most of the reads matched genomic regions that included miRNA, scRNA, snRNA, snoRNA, tRNA, exons, introns and small RNA pseudogenes.

miRNAs play important roles in crucial biological processes, such as cell proliferation, differentiation, and apoptosis^16^, and the dysregulation of miRNAs has been observed in the initiation and progression of a variety of human malignancies^17^,^18^,^19^,^20^,^21^. miRNAs are also epigenetic regulators through the inhibition of protein translation in response to or through the induction of the degradation of targeted mRNA transcripts^22^,^23^. Here, we investigated the association between LIX1L and miRNAs in HEK-293FLG-LIX1L cells. When we examined the differences in expression between HEK-293FLG and HEK-293FLG-LIX1L cells, differentially expressed miRNAs were observed. We identified 158 and 187 miRNAs upregulated in HEK-293FLG-LIX1L cells using AC and Z tests, respectively (*p* ≤ 0.05). Moreover, we identified 267 miRNAs upregulated more than two-fold in the HEK-293FLG-LIX1L cells (*p* ≤ 0.05).

To minimize the risk of false positives, the predictions of each analysis (AC test, Z test and fold-change) were filtered using the scores in the top 10%. The 17 miRNAs with strong prediction scores from at least two analyses were labeled as potential targets for LIX1L (Table 3). The miRNA from both the 5p and 3p strands was detected among each group of significant miRNAs.

### Genes targeted through the identified miRNAs and the LIX1L-induced apoptosis pathway

The assessment of the differences in target gene expression for the miRNAs between HEK-293FLG and HEK-293FLG-LIX1L cells revealed several differentially expressed potential target genes. In the AC test, 1,018, 2,226 and 2,798 target genes were upregulated in HEK-293FLG-LIX1L cells compared with HEK-293FLG cells using three databases (PITA, TargetScan and microRNA.org, respectively). In the Z test, 935, 1,980 and 2,445 target genes were respectively identified using these three databases. Moreover, 576, 1,697 and 1,485 target genes were identified using these three databases to examine fold-changes (PITA, TargetScan and microRNA.org, respectively) (Supplementary Table 2). To investigate the target genes for the 17 miRNAs identified in the AC test, Z test and fold-change analysis, target gene predictions using the Strand NGS were first performed using the three databases (PITA, TargetScan, and microRNA.org). A total of 51, 112, and 140 targets (AC-test, Z test and fold-change, respectively) scoring in the top 5% of each prediction by at least two different programs were identified as the 17 miRNA target genes.

**Figure 5:**
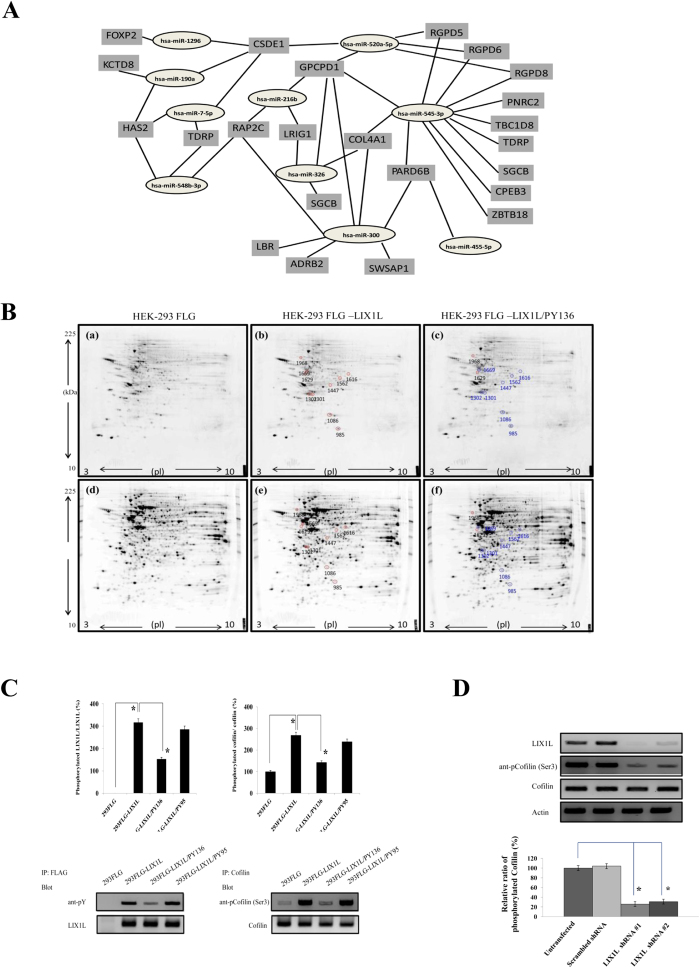
Prediction of target genes for similarly regulated miRNAs and Cofilin phosphorylation through LIX1L. (**A**) The results of common targets for similarly upregulated miRNAs suggested that there was coordination between the miRNAs. (**B**) 2-DE gel images showing the protein expression in HEK-293FLG [(a) and (d)], HEK-293FLG-LIX1L [(b) and (e)] and HEK-293FLG-LIX1L/PY136 [(c) and (f)] cells. The protein samples were loaded onto nonlinear IPG strips (pH 3–10, 17 cm) in an IEF cell and separated through 12% SDS-PAGE. The protein spots were visualized through Pro-Q Diamond phosphoprotein gel staining (upper panels) and SYPRO Ruby protein gel staining (bottom panels). The numbers represent the spot numbers for the detected proteins, and the circles indicate the differentially expressed proteins in HEK-293FLG-LIX1L cells compared with the HEK-293FLG and HEK-293FLG-LIX1L/PY136 cells (red and blue circles indicate up- and downregulation, respectively). Representative images are shown. D/S ratio (phosphorylation index): (%Vol of spot using Pro-Q Diamond stain)/(%Vol of spot using SYPRO Ruby stain) obtained using the Image Master Platinum (GE) software program. (**C**) Western blot analysis of proteins extracted from HEK-293FLG and HEK-293FLG-LIX1L cells treated with or without PY136 (25 μM) or PY95 (25 μM). The ratio of phosphorylated LIX1L and Cofilin to total LIX1L and Cofilin, respectively (upper panels). *p *<* *0.01*. Representative blots from HEK-293FLG and HEK-293FLG-LIX1L cell lines are shown (bottom panels). The cropped blots were run under the same experimental conditions. (**D**) The knockdown of LIX1L protein using *LIX1L* shRNA-#1 or -#2 reduced Cofilin phosphorylation levels in MKN45 cells (bottom panel). The basal levels of Cofilin phosphorylation are expressed as 100%. *p *<* *0.01*. Western blotting results representing three independent experiments are shown (upper panels). Actin was included as a loading control. The blots were run under the same experimental conditions.

Moreover, several miRNAs shared common targets and were similarly upregulated (Fig. 5A). For example, has-miRNA-520a-5p, −300, −216b, and −326 share several common targets [*Glycerophosphocholine phosphodiesterase 1* (*GPCPD1*), *Leucine-rich repeats and immunoglobulin-like domains protein 1* (*LRIG1*) and *Collagen, type IV, alpha 1* (*COL4A1*)]. has-miRNA-190a, −548b-3p, −7–5p and −1296 also shared several common targets [*Cold shock domain containing E1* (*CSDE1*), *Hyaluronan synthase 2* (*HAS2*) and *Testis development related protein* (*TDRP*)]. These putative genes might be associated with LIX1L-mediated cell proliferation.

Moreover, we examined whether the overexpression of LIX1L, as an oncogene, affected the total expression and phosphorylation of proteins in HEK-293 cells. Initially, to determine whether LIX1L affects protein phosphorylation and assess whether this protein is involved in cancer cell proliferation, the protein spots were visualized using Pro-Q Diamond phosphoprotein gel staining (Fig. 5B). The differentially expressed phosphorylated proteins were subsequently identified using Image Master Platinum (GE) software (Table 4). Interestingly, among these proteins, a significant change in phosphorylation was observed for Cofilin (spot 985), which was identified using MALDI-TOF/TOF mass spectrometry and the NCBInr database. Briefly, Cofilin phosphorylation was increased in HEK-293FLG-LIX1L cells compared with HEK-293FLG cells, while Cofilin phosphorylation was decreased in HEK-293FLG-LIX1L cells treated with PY136 (HEK-293FLG-LIX1L/PY136 cells) compared with HEK-293FLG-LIX1L cells.

To confirm the results of the 2-DE and Pro-Q Diamond phosphoprotein gel staining analyses, Cofilin phosphorylation was examined in HEK-293FLG, HEK-293FLG-LIX1L HEK-293FLG-LIX1L/PY136 and HEK-293FLG-LIX1L/PY95 cells using immunoblotting (Fig. 5C). Phosphorylated Cofilin levels were increased in HEK-293FLG-LIX1L cells compared with HEK-293FLG cells, while phosphorylated Cofilin levels decreased after treatment with PY136. Moreover, to confirm the role of LIX1L in Cofilin phosphorylation, we investigated Cofilin phosphorylation in MKN45 cells after LIX1L knockdown using *LIX1L* shRNAs. As shown in Fig. 5D, *LIX1L* knockdown decreased Cofilin phosphorylation (Ser3) in MKN45 cells. These results showed that LIX1L regulated the phosphorylation of Cofilin, and PY136 treatment inhibited both Cofilin and LIX1L phosphorylation.

### Protein tyrosine kinase assay for Tyr136 of LIX1L

We also identified the tyrosine kinase for the phosphorylation of Tyr^136^ of LIX1L using a protein kinase assay. In this assay, two substrates (the [Y136] wild type (WT) peptide and the [F136] mutated peptide, in which the [Y136] site is mutated to a phenylalanine) were used. The profiling data for the [Y136] WT peptide substrate for various protein tyrosine kinases using a radiometric assay method showed strong phosphorylation (ranging from 42,000 to 124,000 cpm) with seven protein kinases, including ROS1, HCK, ABL1, ABL2, JAK3, LCK and TYR03 (Table 5). The protein kinase ROS1 showed the highest counts at 124,391 cpm. In comparison, the counts for the [F136] MT peptide for these same seven kinases were low to moderate, and differences in the counts between the two peptides provided a %CFC in the 67–93% range. All of the other kinases monitored for the [Y136] WT peptide showed a spectrum of counts ranging from zero to 24,000 cpm. In comparison, the highest counts for the [F136] MT peptide substrate were observed with ROS1, ABL1 and ABL2, with counts from 18,000 to 24,000 cpm. The counts for the other kinases for the [F136] MT peptide ranged from zero up to 12,500 cpm. Because the [Y136] site was mutated to a phenylalanine in this peptide, the counts observed indicate the level of phosphorylation at the [Y139] site only. The difference in counts between the [Y136] WT and [F136] MT indicate the level of phosphorylation of each kinase specifically at the [Y136] site.

**Figure 6:**
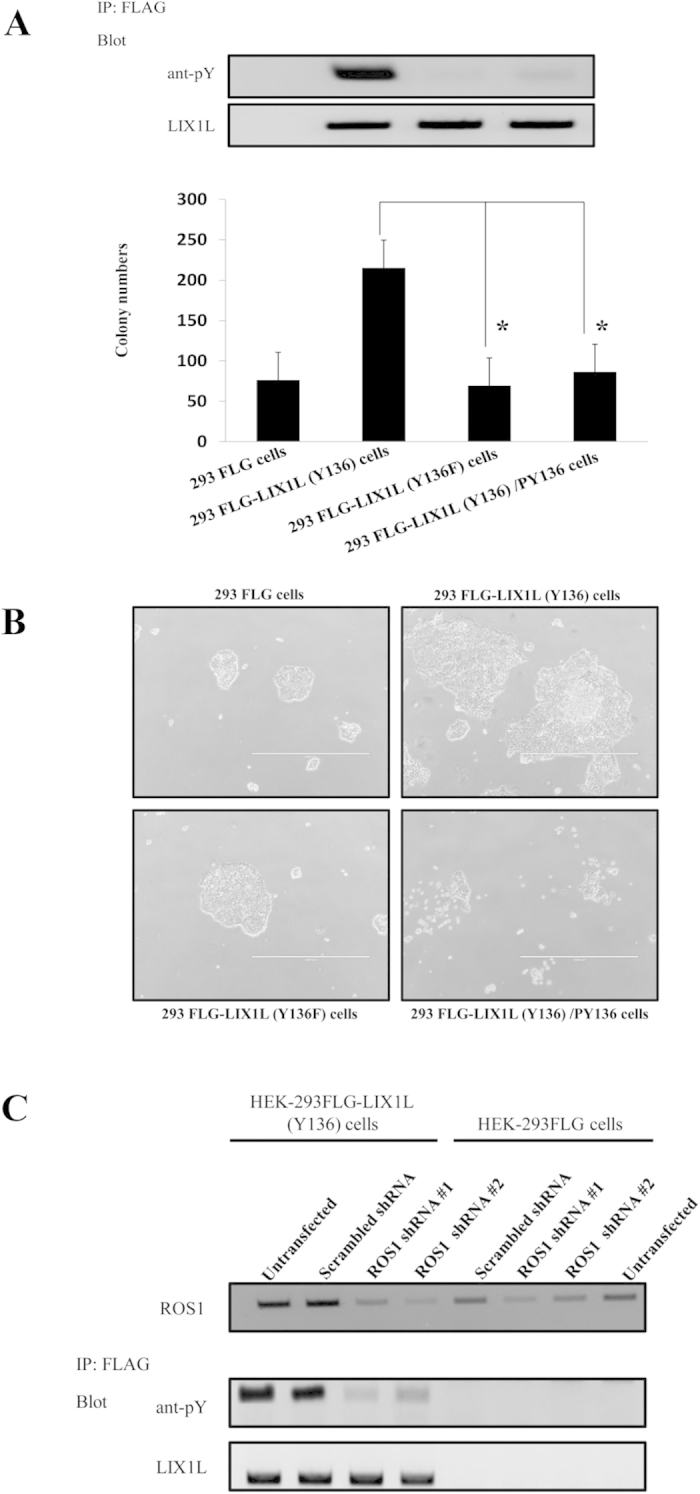
Phosphorylated LIX1L (Y136) promotes cell proliferation. (**A**) The HEK-293FLG, HEK-293FLG-LIX1L (Y136) and HEK-293FLG-LIX1L (Y136F) cells were cultured in semisolid methylcellulose media. The HEK-293FLG-LIX1L (Y136) cells were left untreated or were treated with PY136 (25 μM). After 14 days in culture, colony formation was analyzed, and the cells were viewed using phase-contrast microscopy. The colonies formed from each cell type (3 × 10^2^ to 5 × 10^2^ cells/plate) were counted following plating in semisolid methylcellulose media and the phosphorylation levels of LIX1L were analyzed through western blotting. The phosphorylation level of LIX1L was decreased compared with that in LIX1L (Y136) cells. The blots were run under the same experimental conditions. These data are shown as the mean ± SD for independent experiments. *p *<* *0.01*. (**B**) The representative microscopic images of each colony are shown. Original magnification 4x. (**C**) HEK-293FLG and HEK-293FLG-LIX1L (Y136) cells were transfected with scrambled shRNA, *ROS1* shRNA-#1 or -#2, and the phosphorylation level of LIX1L was assessed. The blots were run under the same experimental conditions.

Moreover, the colony-formation counts and the phosphorylation levels were decreased in HEK-293FLG-LIX1L (Y136F) cells (Fig. 6A,B). The colony counts of HEK-293FLG-LIX1L (Y136F) cells were lower than those of HEK-293FLG and HEK-293FLG-LIX1L (Y136) cells. The level of phosphorylated LIX1L was also decreased in HEK-293FLG-LIX1L (Y136F) cells compared with HEK-293FLG-LIX1L (Y136) cells. Moreover, the knockdown of ROS1, the putative LIX1L kinase, reduced LIX1L phosphorylation in HEK-293FLG-LIX1L (Y136) cells. Both HEK-293FLG and HEK-293FLG-LIX1L (Y136) cells were transfected with *ROS1* shRNA-#1 or -#2, and the phosphorylation level of LIX1L was assessed on day 3 post-transfection. The *ROS1* shRNA-#1 or -#2 mediated *ROS1* mRNA expression knockdown by 77% and 71%, respectively (data not shown). ROS1 knockdown in HEK-293FLG-LIX1L (Y136) cells significantly reduced LIX1L phosphorylation compared with both untreated HEK-293FLG cells and HEK-293FLG-LIX1L cells transfected with scrambled shRNA (Fig. 6C). These results showed that ROS1 is the main regulator of Tyr^136^ phosphorylation in LIX1L, and the phosphorylation of Tyr^136^ in LIX1L plays an important role in cancer cell proliferation.

## Discussion

Cancer is a major cause of death worldwide. Currently, the common treatments for cancer include chemotherapy, surgery and radiotherapy. However, cancer treatment remains unsatisfactory because of the high failure rates and adverse effects of these treatments. Recent findings in cancer cell biology, based on the knowledge derived from molecular, cellular and systems biology studies of cancer progression, have led to the development of novel targeted anticancer drugs^24^,^25^,^26^. The application of several novel compounds in clinical trials has demonstrated that these treatments are promising anticancer therapies^27^,^28^. However, the successful treatment of cancer with targeted therapies remains the exception, and this treatment is limited to malignancies for which specific genetic, epigenetic or metabolic causes have been identified. Specific proteins expressed in cancer cells selectively induce apoptosis in tumor cells. This concept of tumor-selective cell death has attracted substantial research attention worldwide, with promising results^4^. In the present study, we demonstrated that the LIX1L protein is preferentially expressed in human cancer cells, regardless of the cancer type, and therefore this protein could be targeted for therapeutic effects in a wide range of cancer types.

LIX1L is a putative RBP, and these genes play important roles in post-transcriptional gene regulation, but examples relevant to human disease remain unknown. An investigation of the mechanism(s) by which the LIX1L protein promotes cancer cell proliferation has highlighted the presence of common pathways shared among different tumor types. In the present study, several potential mechanisms for LIX1L-induced cancer growth or progression were identified, including the promotion of cell proliferation, interactions with other proteins, binding of miRNAs, targeting gene expression and the formation of complexes leading to the phosphorylation of various proteins.

LIX1L is expressed in various cancer tissues and cancer cell lines. Interestingly, both the knockdown of LIX1L using shRNA and the reduction of phosphorylated LIX1L using PY136 peptide inhibited cancer cell proliferation *in vitro* and *in vivo* and decreased phosphorylated Cofilin. The increased expression and phosphorylation of Cofilin might play a role in bladder cancer progression^29^. LIX1L might affect Cofilin phosphorylation to promote cancer cell proliferation. We also identified LIX1L-associated putative miRNAs (has-miRNA-520a-5p, −300, −216b, −326, −190a, −548b-3p, −7–5p and −1296). miRNA-520a-5p was highly expressed in mature ovarian teratoma tissues^30^, and the function of this molecule remains unknown. miRNA-300 is upregulated in the liver of methionine- and choline-deficient (MCD)-fed mice^31^, and the function of this molecule is also unknown. miRNA-216b inhibits the proliferation, migration and invasion of HCC through the regulation of insulin-like growth factor 2 mRNA-binding protein 2 (IGF2BP2)^32^. miR-326 has tumor-suppressive properties and is downregulated in glioblastoma samples^33^. mR-190a is a metastasis suppressor, and the molecular mechanism through which ERα regulates miR-190a expression remains unknown^34^. miR-548b-5p as noninvasive biomarker is downregulated in malignant astrocytomas^35^. miRNA-7-5p, a novel tumor suppressor in melanoma, acts at least in part through the inhibition of IRS-2 expression and oncogenic Akt signaling^36^. miR-1296 expression was significantly reduced in prostate tumors compared with benign prostate hypertrophy tissues. The transfection of miR-1296 in PC3 prostate cancer cells decreased the expression of *MCM2* mRNA and protein^37^. Thus, in human cancers, certain miRNAs might have an oncogenic function, reflecting the overexpression of these molecules in malignant tissues, whereas other miRNAs might act as tumor suppressor genes, reflecting the decreased expression observed for these molecules. However, the direct interaction LIX1L with these miRNAs has not been confirmed. Therefore, further studies are needed to assess this potential interaction. The findings of the present study provide the first evidence of an altered miRNA profile for LIX1L-expressing HEK293 cells and differentially expressed miRNAs, which, if validated in future studies, might be essential in the pathogenesis of LIX1L-expressing cancer cells. We also identified putative miRNA-targeted genes (*GPCPD1, LRIG1, COL4A1, CSDE1, HAS2* and *TDRP*). The *COL4A1* gene has been associated with breast cancer progression^38^, and this gene is overexpressed in glioblastoma^39^. The silencing of the CSDE1 gene significantly increases the sensitivity to platinum based chemotherapy in ovarian cancer cell lines^40^. The *HAS2* gene promotes breast cancer progression^41^; in contrast, the *LRIG1* gene enhances radiosensitivity in glioblastoma cells through the attenuation of the EGFR/Akt signaling pathway^42^. *GPCPD1* and *TDRP* genes have not been associated with cancer cell proliferation. These miRNA-mRNA target analyses suggests the coordinated interplay between several miRNAs in the regulation of target genes that might play a role in the LIX1L-mediated cell proliferation, and the roles of these genes in cancer cells require further validation.

ROS1 (ROS1 proto-oncogene receptor tyrosine kinase) is activated through chromosomal rearrangement in a variety of human cancers, including glioblastoma multiforme, non–small-cell lung cancer (NSCLC), cholangiocarcinoma, gastric cancer, and ovarian cancer^43^,^44^,^45^,^46^,^47^. However, the function of WT ROS1 and a ligand for the receptor remain unknown. The lack of a known ligand has also impeded the investigation of down-stream signaling activated through WT ROS1^48^. In the present study, we identified ROS1 as a putative regulator of LIX1L phosphorylation. Indeed, LIX1L is a downstream signaling molecule activated through WT ROS1.

Therefore, the discovery of an inhibitor of LIX1L, such as shRNA, homeodomain peptides and inhibitors of specific ROS1 kinases would be useful to identify potential tumor-specific drugs for LIX1L-expressing cancer cells. In the present study, we revealed that LIX1L led to common changes in cancer cells, such as signaling activation. We showed that PY136 inhibited cancer cell proliferation by inhibiting the phosphorylation of Tyr^136^ of the LIX1L protein *in vitro* and *in vivo*, and ROS1 phosphorylated LIX1L protein to result in cell proliferation. Thus, the results of the present study showed that LIX1L and the modifying kinase, ROS1, can be exploited as potential targets in LIX1L-expressing cancers.

## Materials and Methods

### Reagents

A Rabbit anti-LIX1L polyclonal antibody (Abnova, Taipei, Taiwan), mouse anti-FLAG monoclonal antibody (Sigma-Aldrich, St. Louis, MO, USA), rabbit anti-phosphothreonine polyclonal antibody (Abcam, Cambridge, MA, USA), rabbit anti-phosphoserine polyclonal antibody (Abcam), rabbit anti-phosphotyrosine polyclonal antibody (Abcam), rabbit anti-RIOK1 polyclonal antibody (Santa Cruz Biotechnology, Dallas, TX, USA), rabbit anti-nucleolin polyclonal antibody (Origene, Rockville, MD, USA), rabbit anti-PABPC4 polyclonal antibody (Abnova), rabbit anti-Cofilin polyclonal antibody (Cell Signaling Technology (CST), Beverly, MA, USA), rabbit anti-phospho-Cofilin (Ser 3) polyclonal antibody (CST), rabbit anti-α Tubulin polyclonal antibody (Abcam), rabbit anti-Lamin A + C monoclonal antibody (Abcam), rabbit anti-ROS1 monoclonal antibody (CST) and anti-Actin antibody (Abcam) were used in the present study.

### Tissue immunohistochemistry (IHC)

All tissues were surgically collected under the supervision of an experienced pathologist. The LIX1L expression was measured using IHC. IHC staining was performed using a rabbit polyclonal LIX1L antibody (diluted 1:500, Abnova. Taipei, Taiwan). PBS was used to replace the primary antibody in negative controls. LIX1L was primarily located in the cytoplasm, with weaker staining in the nucleus. Normal tissues were generally negative. Tissues were evaluated and scored when the cell cytoplasmic and/or nuclear reactivity was observed, but no relevant clinical cut-off point or standard evaluation method has previously been reported for LIX1L. This study was approved through the Institutional Ethics Commission of Hamamatsu University School of Medicine, and the methods were performed in accordance with the approved guidelines.

In the present study, three independent observers randomly and blindly selected and counted one hundred cells from five representative fields of each section. The positive percentage of the counted cells was graded semi-quantitatively according to a four-tier scoring system: negative (0); 0–5%, weakly positive (1); 6–25%, moderately positive (2); 26–50%; and 51–100%, strongly positive (3). All subjects provided informed consent to participate in this study. This study was performed in accordance with the approved guidelines through the Ethics Committee of Hamamatsu University School of Medicine. The methods were performed in accordance with the approved guidelines.

### Cells and cell cultures

Two human gastric cancer cell lines, NUGC-4 and MKN45, were purchased from the RIKEN BioResource Center (Tsukuba, Japan). The KATOIII and OCUM-1 human gastric cancer cell lines were obtained from the Japanese Collection of Research Bioresources (Tokyo, Japan). All cells were cultured in RPMI 1640 containing 10% heat-inactivated fetal bovine serum (FBS), 2 mM L-glutamine, 100 μg/ml streptomycin and 200 U/ml penicillin (GIBCO-BRL, Gaithersburg, MD, USA).

### Stable expression of human LIX1L in the Flp-In-293 systems

The pcDNA5/FRT plasmid containing the full length human *LIX1L* cDNA was transfected into Flp-In-293 cells in combination with pOG44 according to the manufacturer’s instructions (Invitrogen). After recovery, the cells were selected in 100 μg/ml hygromycin B for approximately three to four weeks. The Flp-In-293 cells expressing LIX1L (HEK-293FLG-LIX1L cells) were maintained in complete DMEM containing 10% FBS, L-glutamine and 100 μg/ml of hygromycin B for subsequent experiments.

### RT-PCR and quantitative real-time PCR (QRT-PCR)

Total RNA was extracted from cells using the RNeasy system (Qiagen, Tokyo, Japan), and 2 μg of RNA was reverse transcribed using a first-strand cDNA synthesis kit (Roche Life Science Inc., Indianapolis, IN, USA). PCR was performed using a DNA thermal cycler (model PTC 200; MJ Research, Watertown, MA, USA). The following sense and anti-sense oligonucleotide sequences for each primer were used: *LIX1L*, 5′-GGGAGGGGCACTCTCCGAGC-3′ and 5′-GCGAAGCTCCTCACCACGGC-3′; *GAPDH*, 5′-GAACAGCAACGAGTACCGGGTA-3′ and 5′-CCCATGGCCTTGACCAAGGAG-3′. The PCR conditions for *LIX1L* and *GAPDH* included 28 cycles of denaturation at 96 °C for 30 sec followed by annealing at 56 °C for 30 sec and extension at 72 °C for 30 sec. All RT-PCR experiments were performed in duplicate. QRT-PCR was performed using SYBR green dye and an ABI PRISM 7700 sequence detector (Perkin-Elmer/Applied Biosystems, Foster City, CA, USA).

### Plasmids and RNA interference

Full-length cDNAs encoding human *LIX1L* were obtained through RT-PCR using human bone marrow cDNA (BD Biosciences Clontech, Palo Alto, CA, USA) as a template and cloned into the eukaryotic expression vector pcDNA3.1/V5-His (Invitrogen, Carlsbad, CA). The sequence of recombinant *LIX1L* cDNA was verified using automated sequencing.

The vectors used for RNA interference (RNAi) specific for human *LIX1L* and *ROS1* were constructed based on the piGENE PUR hU6 vector (iGENE Therapeutics, Tsukuba, Japan), according to the manufacturer’s instructions. Briefly, to produce shRNA-encoding vectors, the hU6 promoter and the hairpin construct were fused using a universal 5′ hU6 primer and a 3′ primer that included the siRNA targeting *LIX1L* mRNA sequences. The following targeting sequences were used: *LIX1L* shRNA #1, 5′-AGGAGGTGTTGGCTCATTATT-3′; *LIX1L* shRNA #2, 5′-AGTCCCGTGGTGCTGACTTAA-3′; *ROS1* shRNA #1, 5′-ACCTTTGATTAGGAATATTGAGA-3′; and *ROS1* shRNA #2, 5′-GGCTTTATCAAAGAATGTATTTC-3′. The scrambled shRNA sequence 5′-GGACGAACCTGCTGAGATAT-3′ was used as a control. The vectors were transfected into cells using the Lipofectamine 2000 kit (Life Technologies, Gaithersburg, MD, USA), according to the manufacturer’s instructions. The transfection procedure was repeated 12 h after the first transfection, and the cells were harvested at 48 and 72 h after the initial transfection. The knockdown efficiency was consistently 65% to 80%, determined through RT-PCR measurement of *LIX1L* and *ROS1* mRNA.

### Peptide design, synthesis and internalization

The homeodomain peptides were designed for the eleven serine, three threonine and three tyrosine phosphorylation sites predicted using the NetPhosK 2.0 server (http://www.cbs.dtu.dk/services/NetPhos/)^49^. The sequences of homeodomain peptides for LIX1L are shown in Table 1. The peptides showed a purity greater than 98% and were synthesized and labeled (FAM) at the TORAY Research Center (Kanagawa, Japan). The peptides were generated after coupling the TAT sequence (YGRKKRRQRRR), followed by receptor-independent cell entry and an assessment of the amino acid positions of human LIX1L through an ε-aminocaproic acid or FAM, respectively. A control peptide (Pcon) (YGRKKKRRQRRR-NH_2_) and the chimeric peptides were synthesized and purified using reverse-phase high performance liquid chromatography (HPLC) (Toray Research Center, Otsu, Japan). The peptides were dissolved in filtered sterile PBS at a concentration of 10 mM and stored in aliquots at −80 °C. The cells were incubated and added to wells coated with the peptide. After incubation for 3 h, the cells were visualized using phase-contrast and fluorescence microscopy and analyzed using flow cytometry. The nuclei were stained using Hoechst 33342 (Sigma-Aldrich).

### Cell proliferation and viability assay, and the caspase activity assay

MKN45, OCUM, KATOIII and NUGC-4 cells were untreated or transfected with *LIX1L* shRNA-#1 or -#2 or scrambled shRNA. After a 3-day incubation, the cells were seeded onto 96-well, flat-bottomed microplates at a density of 1 × 10^4^ cells per well. Cells grown in complete medium without transfection were used as controls.

Cell proliferation was assessed after counting the viable cells on the indicated days using trypan blue (Sigma-Aldrich) exclusion. The number of nonviable cells was determined after counting the live cells that did not take up the trypan blue using a hemocytometer. To assess the cell viability, an MTT assay was performed. The cells were seeded onto 96-well, flat-bottomed microplates at a density of 5 × 10^4^ per well. The cells were transfected with *LIX1L* shRNA-#1, -#2 or scrambled shRNA, while untransfected cells were used as an additional control. After a 3-day incubation, 10 μl of 3-(4,5-dimethylthiazol-2-yl)- 2,5-diphenyltetrazolium bromide (MTT) solution (Sigma-Aldrich, St. Louis, MO, USA) was added to each well at a final concentration of 1 mg/ml. The cells grown in complete medium alone were used as controls. After incubation at 37 °C for 4 h, the absorbance was measured at a wavelength of 570 nm with a microplate reader. The BrdU Labeling and Detection Kit III (Roche Life Science Inc.) was used to quantify cell proliferation accordance to the manufacturer’s instructions. The fluorescence was measured using a microplate reader, and the values of the BrdU assay obtained for the untreated control cells were set at 100%. Caspase activity was determined using the Caspase-Glo 3/7 and 9 Assay Kit (Promega, Madison, WI, USA) according to the manufacturer’s instructions. Briefly, after treatment, floating and attached cells in 6-well plates were harvested and suspended in culture medium for detection. The cells were counted, and 100 μl of Caspase-Glo 3/7 or Caspase-Glo 9 reagent was added to each well of a black 96-well plate containing samples or blank medium. After mixing the contents of the wells using a plate shaker and incubation at room temperature, the fluorescence was measured using a microplate reader. Each data point was performed in triplicate, and the results are reported as the mean count ± SD.

### Cell cycle analysis

The cellular DNA content was analyzed using propidium iodide (PI; Sigma-Aldrich) staining. The cells were stained with 50 μg/ml PI on day 3 post-transfection. The relative DNA content per cell was measured using flow cytometry and an Epics Elite flow cytometer (Coulter Immunotech, Marseille, France). The percentage of cells in the G1, S and G2/M phases was calculated using the ModFit program (Becton Dickinson, San Jose, CA, USA).

### Preparation of whole, nuclear and cytosolic extracts, immunoprecipitation and western blot analysis

Proteins extracted from frozen surgical specimens (gastric cancer #1 and #2; colon cancer #3 and #4; lung cancer #5 and #6) and transfected cells were immunoblotted with different antibodies. Nuclear and cytosolic proteins were extracted using Nuclear/Cytosolic Fraction Kit according to the manufacturer’s instructions (Cell Biolabs, Inc., San Diego, CA, USA). Briefly, the cells were homogenized in ice-cold cytosol extraction buffer, and cell lysis reagent was added. The homogenate was centrifuged at 800 × *g*, and the supernatant was separated and saved as a cytosolic fraction. The resultant nuclear fraction was washed several times with nuclear extraction buffer and incubated on ice for 30 min. The mixture was centrifuged at 14,000 × *g* for 30 min. The supernatant was separated and stored at −80 °C until further use. The anti-α Tubulin and anti-Lamin A + C antibodies were used to identify the cytosolic and nuclear fractions, respectively. To detect the proteins associated with the LIX1L protein, immunoprecipitation was performed using an anti-FLAG or anti-LIX1L antibody. A western blot analysis was performed on 12% SDS-PAGE, and the bands were visualized after silver staining and immunoblotting using enhanced chemiluminescence reagents (Amersham Pharmacia, UK) according to a standard protocol.

### Tumor xenograft studies

A total of 1 × 10^6^ or 1 × 10^7^ tumor cells were subcutaneously injected in the hind flank of a nude mouse. Tumor-bearing mice were size-matched and divided into groups of four mice per group with a tumor volume of 100 mm^3^ to 150 mm^3^, and drug treatment was initiated at this time. A dose of 100 mg/kg of PY136 was subcutaneously injected twice/week for two weeks (n = 3). The vehicle was PBS (n = 3). The tumor volumes were estimated twice weekly based on two-dimensional caliper measurements obtained using the prolate ellipsoid equation *v* = (*lw*^2^/2), where *v* = volume (mm^3^), *l* = length (mm) and *w* = width (mm), for three weeks. The tumor growth inhibition represents the percentage difference in the volume between the treated and control tumors. All animal experiments were performed in accordance with the guidelines for the Care and Use of Laboratory Animals of the US National Institutes of Health. The current study protocol was also approved through the Institutional Animal Care and Use Committee of Hamamatsu University School of Medicine, and the methods were performed in accordance with the approved guidelines.

### Soft agar colony formation assay

The stable HEK-293FLG-LIX1L cells were subjected to soft agar assays using six-well plates containing semisolid medium (10% FBS-DMEM, 0.4% agar) on a base layer of medium (10% FBS-DMEM, 0.6% agar). The colonies were enumerated under light microscopy (Zeiss, Munich, Germany) following incubation for 14 days at 37 °C at 5% CO_2_. Three independent experiments were performed.

### Protein extraction and quantification

At 12 h after treatment with or without pY136, the HEK-293FLG-LIX1L cells were harvested, washed and immediately homogenized in a lysis buffer (5 M urea, 2 M thiourea, 2% CHAPS, 2% IPG buffer [pH 3–10], 1% DTT). Each sample was subsequently centrifuged at 22,000 × *g* for 0.5 h. The supernatants were carefully removed, and the protein concentrations were analyzed in triplicate using a 2D Quant Kit (GE Healthcare, Piscataway, NJ, USA). The supernatant samples were subsequently aliquoted and stored at −80 °C until further analysis.

### Two-dimensional gel electrophoresis (2-DE) and MALDI-TOF/TOF MS

For each sample, 500 mg of protein was mixed with rehydration buffer (5 M urea, 2 M thiourea, 100 mM DTT, 2% CHAPS, 0.5%, 2% IPG buffer [pH 3–10], and 0.2% Pharmalyte pH 3–10) to a final volume of 200 ml and was incubated for 1 h at room temperature. The samples were passively rehydrated onto 18-cm immobilized pH gradient (IPG) strips (pH 3–10; GE Biosciences). Isoelectric focusing was performed using a CoolPhoreStar IPG-IEF System (GE Biosciences). The proteins were isoelectrically focused at 500 V for 1 min, slowly ramped up to 3500 V for 90 min and subsequently maintained at 3500 V for 5.5 h. Each IPG strip was stored at −80 °C until second-dimension processing. Each IPG strip was placed on top of a large format 9–18% SDS-polyacrylamide concentration gradient gel (20 cm × 21 cm), along with a broad range of protein molecular weight markers (V8491, Promega) and subjected to 2DE at 80 V per gel for 17 h using an ANDERSON ISO DALT electrophoresis system (HOEFER). The gels were subsequently fixed in methanol/acetic acid (40%/10% in dH_2_O) for 1 h at room temperature and incubated in SYPRO Ruby (Bio-Rad laboratories, USA) for 16 h at room temperature on a rocking platform. The gels were destained for 1 h in methanol/ acetic acid (10%/7% in dH_2_O), imaged using a Molecular Imager FX imager (Bio-Rad) at 100 nm resolution and analyzed using ImageMaster 2D platinum (GE). The computer program identified protein spots from the digital images of the gel.

Western blotting and in-gel digestion were performed prior to ESI-MS/MS analysis with MALDI-TOF mass spectrometry. The peptides were desalted using the ZipTipCm-C8 device according to the manufacturer’s instructions (Millipore Corporation, Billerica, MA, USA). The peptide samples were mixed with CHCA matrix solution and analyzed using a MALDI-TOF/TOF mass spectrometer (ultrafleXtreme MALDI-TOF/TOF mass spectrometer, Bruker Daltonics, Bremen, Germany). The search parameters included trypsin, one missed cleavage, cut-off individual ion scores >20 and extensive homology *p* < 0.05. The spectra were processed and analyzed using the Global Protein Server Explorer 3.0 software program (Applied Biosystems). The internal Mascot software program (Matrix Science Ltd., London, UK) was used for matching the MS and MS/MS data against database information. The resulting data were surveyed against human databases downloaded from the NCBInr homepage.

### RNA binding protein immunoprecipitation and RNA extraction

The HEK-293FLG and HEK-293FLG-LIX1L cells were grown in complete DMEM medium containing 10% FBS, L-glutamine and 100 μg/ml of hygromycin B. The LIX1L binding proteins were pulled down using the anti-Flag antibody, and the RNA was extracted from the cells using the Magna RIP RNA-binding Protein Immunoprecipitation Kit (Merck Millipore, Billerica, MA, USA) according to the manufacturer’s instructions. Total RNA, small RNA and the miRNA abundance and quality were determined after extraction using a Qubit 2.0 Fluorometer (Life Technologies, Carlsbad, CA, USA) and an Agilent 2100 Bioanalyzer (Agilent Technologies, Santa Clara, CA, USA), respectively.

### Preparation of miRNA libraries for next-generation sequencing

The small RNA libraries used for NGS were prepared from RNA samples obtained from both HEK-293FLG and HEK-293FLG-LIX1L cells. Each library was prepared from the 20 to 30 nucleotide RNA fraction. These samples were subjected to RNA fragmentation, adaptor hybridization and ligation, PCR amplification and emulsion PCR using the Ion Total RNA-Seq Kit v2 (Life Technologies) according to the manufacturer’s instructions. The abundance of the libraries was measured using a Qubit 2.0 fluorometer (Life Technologies). The size of the products contained in the libraries was assessed using an Agilent 2100 Bioanalyzer (Agilent Technologies). The libraries were sequenced using an Ion Torrent semiconductor sequencer (Life Technologies) according to the manufacturer’s standard protocols.

### Analysis of the microRNA next-generation sequencing data

Small RNA sequencing data were processed from raw FASTQ files and analyzed using the Strand NGS program (Strand Genomics Co.)^50^. After filtering the read data, the expression was quantified using the Strand NGS program (Strand Genomics, Inc.), which quantified the active regions using only reads precisely matching the active region boundaries, with a normalized algorithm^51^,^52^, based on the baseline to median/mean of all samples.

For the read data after quantification, the known and novel small RNAs were extracted using the Strand NGS program. The extraction of known mature miRNAs was also performed^50^. The extracted expression variations of the small RNAs and miRNAs were analyzed using statistical tests. For the extraction of differences in small RNAs and miRNAs expression between HEK-293FLG and HEK-293FLG-LIX1L cells, the Audic-Claverie Test (AC Test), Z test and fold-change analysis were performed.

### miRNA target prediction and pathway analysis

Potential target genes of the miRNAs were identified and mapped to signaling pathways related to the target genes. We used multiple prediction programs, including PITA^53^, TargetScan^54^ and microRNA.org^55^. To minimize the risk of false positives, the predictions of each program were filtered using only reads scoring within the top 5%. Genes with strong prediction scores for the same miRNA from at least two programs were labeled as potential targets for that miRNA and were used in the pathway analysis. For miRNAs with no target gene shared by multiple programs, genes with prediction scores ranking within the top 1% in any program were used as potential target genes for miRNA.

### Site-directed mutagenesis and plasmid construction

A single amino acid mutation (Y136F; mutated Tyr^136^ to Phe^136^) was generated in LIX1L in a pCMV6-XL4 vector using the QuickChange Site-Directed Mutagenesis Kit (Stratagene, La Jolla, CA, USA) with paired primers (CAGCCCTCCTTTTGTCTGCT and CATAGCAGACAAAAGGAGGG), and the presence of the mutation was confirmed through sequencing.

### Protein tyrosine kinase screening assay

To identify the kinase that phosphorylates residue Tyr^136^ of LIX1L, a screen of 74 recombinant protein tyrosine kinases was performed at KINEXUS (Vancouver, Canada). Briefly, the peptides [Y136] WT (PSNSPPYVCYKK) or [F136] MT (PSNSPPFVCYKK) were mixed with individual protein kinases in the presence of [γ-^33^P] ATP (Perkin Elmer). After removing unreacted [γ-^33^P] ATP from the reaction, the radioactivity was quantified in a Trilux scintillation counter (Perkin Elmer). The radioactivity of [Y136] WT was compared with that of [F136] MT. Moreover, a comparison of the differences in counts between these two peptides and the percent change from the control (CFC) was instructive for determining the effects of the mutation on kinase recognition.

### Statistical analyses

The data are representative of at least three experiments with essentially similar results. These results are expressed as the mean ± standard deviations (SD) or standard error of the means as indicated. The statistical analyses of the data were performed using Student’s *t*-test. *P* values < 0.05 were considered significant.

## Additional Information

**How to cite this article**: Nakamura, S. *et al.* Novel roles for LIX1L in promoting cancer cell proliferation through ROS1-mediated LIX1L phosphorylation. *Sci. Rep.* **5**, 13474; doi: 10.1038/srep13474 (2015).

## Supplementary Material

Supplementary Information

## Figures and Tables

**Table 1:**
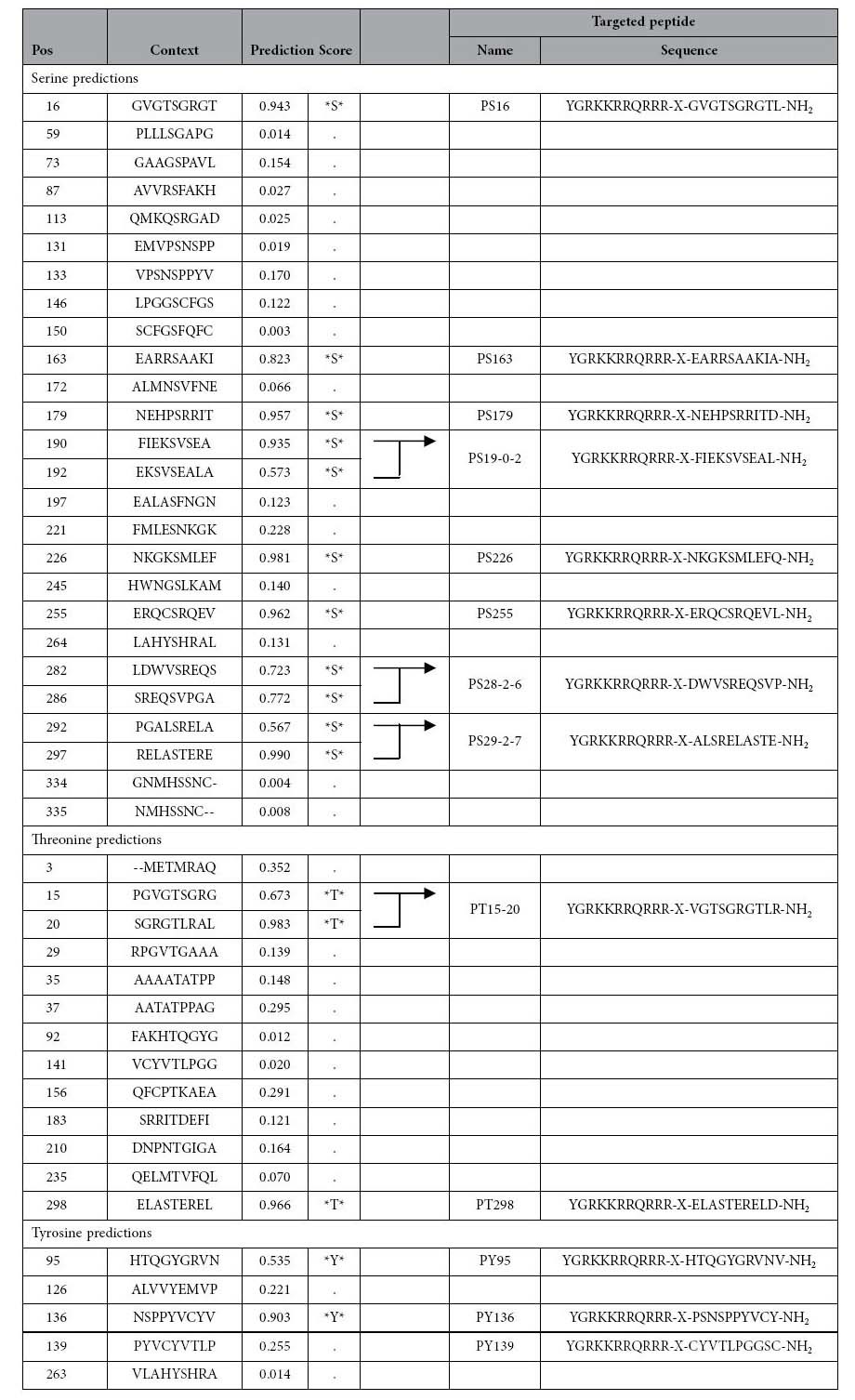
The results of the peptide sequence analysis using the NetPhos 2.0 software program.

Sixteen peptide sequences were recovered and analyzed. The sequences with a high prediction score for each phosphorylation site were selected. Pos; Position. The eight homeodomain peptide sequences targeted serine phosphorylation sites. The two homeodomain peptide sequences targeted threonine phosphorylation sites. The three homeodomain peptide sequences targeted tyrosine phosphorylation sites.

**Table 2:** The identification of LIX1L-associated proteins by MALDI-TOF/TOF mass spectrometry.

SampleName		Protein	gene	MW	Score	Peptide	Coverage	Accession	Note*
C-p89	1	X-ray repair cross-complementing protein 5	XRCC5 G22P2	82,705	218	18	30	P13010	*gi|10863945*
	2	Serine/threonine-protein kinase RIO1	RIOK1	65,583	122	12	19	Q9BRS2	*gi|16549132*
	3	Protein arginine N-methyltransferase 5	PRMT5 HRMT1L5, IBP72, JBP1, SKB1	72,684	61	7	10	O14744	*gi|2323410*
	4	Eukaryotic translation initiation factor 4B	EIF4B	69,151	47	3	6	P23588	*gi|288100*
C-p80	1	78 kDa glucose-regulated protein	HSPA5 GRP78	72,333	617	27	46	P11021	*gi|6470150*
	2	Nucleolin	NCL	76,614	390	24	32	P19338	*gi|189306*
	3	Protein arginine N-methyltransferase 5	PRMT5 HRMT1L5, IBP72, JBP1, SKB1	72,684	234	15	25	O14744	*i|2323410*
	4	Polyadenylate-binding protein 4	PABPC4 APP1, PABP4	70,783	159	15	27	Q13310	*gi|48734702*
	5	Influenza virus NS1A-binding protein	IVNS1ABP ARA3, FLARA3, KIAA0850, NS1, NS1BP	71,729	88	11	19	Q9Y6Y0	*gi|6841374*
	6	Probable ATP-dependent RNA helicase DDX20	DDX20 DP103, GEMIN3	92,241	74	5	7	Q9UHI6	*gi|5359631*
	7	Hemoglobin subunit alpha	HBA1 HBA2	15,258	48	2	17	P69905	*gi|229751*
	8	ATP-dependent RNA helicase DDX3X	DDX3X DBX, DDX3	73,243	42	3	5	O00571	*gi|188036020*

Note* : Accession in Mascot Search Results. The supernatants derived from the cytoplasmic fraction of HEK-293FLG-LIX1L cells treated without RNase were analyzed by MALDI-TOF/TOF mass spectrometry.

**Table 3:** Differential expression of miRNA between the HEK-293FLG and HEK-293FLG-LIX1L cells.

miRNA	AC test	Z test	Fold change
	*p*-value	*p*-value	>=2
*hsa-miR-520a-5p*	3.47E-11	3.47E-11	5.248175
*hsa-miR-300*	1.19E-09	1.19E-09	5.013709
*hsa-miR-190a*	1.34E-07	1.34E-07	4.626686
*hsa-miR-4728-5p*	1.50E-05	1.50E-05	4.096171
*hsa-miR-548b-3p*	1.50E-05	1.50E-05	4.096171
*hsa-miR-216b*	4.89E-05	4.89E-05	3.926246
*hsa-miR-148a-5p*	4.89E-05	4.89E-05	3.926246
*hsa-miR-10a-3p*	1.59E-04	1.59E-04	3.733601
*hsa-miR-545-3p*	0.0010404	9.83E-04	3.733601
*hsa-miR-3662*	5.18E-04	4.56E-05	3.511209
*hsa-miR-3173-5p*	5.18E-04	4.56E-05	3.511209
*hsa-miR-16-1-3p*	5.18E-04	4.56E-05	3.511209
*hsa-miR-34a-3p*	0.0030271	0.002991	3.511209
*hsa-miR-7-5p*	0.0030271	0.002991	3.511209
*hsa-miR-455-5p*	0.0016842	1.98E-04	3.248175
*hsa-miR-1296*	0.0016842	1.98E-04	3.248175
*hsa-miR-326*	0.0016842	1.98E-04	3.248175

*p*-value was adjusted for false discovery rate of 5%.

**Table 4:** The identification of the phosphorylated proteins by 2DE.

GroupID	93FLG			293FLG-LIX1L			Difference ofthe D/S ratio(FLGLIX1L/FLG)	293FLG-LIX1L/PY136		
	Diamond(%Volume)	Pro-Q Ruby(%Volume)	SYPROD/S ratio	Pro-QDiamond(%Volume)	SYPRO Ruby(%Volume)	D/Sratio		Pro-QDiamond(%Volume)	SYPRO Ruby(%Volume)	D/Sratio
985	0.29	0.07	4.15	0.40	0.04	9.78	2.36	0.44	0.05	8.61
1669	0.22	0.08	2.86	0.31	0.06	4.94	1.73	0.38	0.11	3.36
1301	0.32	0.11	2.91	0.44	0.09	4.95	1.70	0.38	0.11	3.56
1562	0.08	0.03	2.60	0.12	0.03	4.31	1.66	0.11	0.04	3.01
1616	0.06	0.09	0.64	0.07	0.06	1.04	1.63	0.05	0.06	0.81
1447	0.06	0.06	1.05	0.07	0.04	1.72	1.63	0.05	0.06	0.87
1302	0.60	0.15	4.10	0.82	0.13	6.24	1.52	0.83	0.18	4.63
1086	0.39	0.60	6.39	0.49	0.05	9.58	1.50	0.47	0.09	5.32

The phosphoprotein spots having significant changes. The supernatants derived from the cytoplasmic fractions of HEK-293FLG, HEK-293FLG-LIX1L and HEK-293FLG-LIX1L/PY136 cells were analyzed by 2DE. Spot#985 indicates cofilin-1, which was identified by MALDI-TOF/TOF mass spectrometry.

**Table 5:** Activity of protein tyrosine kinases listed using the radiometric assay method. [Y136]WT: PSNSPPYVCYKK. [F136]MT: PSNSPPFVCYKK.

Kinases	WT(CPM)	MT(CPM)	Difference(WT-MT)	%CFC
ROS1	124,391	24,140	100,251	81
HCK	77,752	7,469	70,283	90
ABL1	64,612	20,116	44,496	69
ABL2	55,031	18,183	36,848	67
JAK3	50,944	6,172	44,772	88
LCK	46,181	3,251	42,930	93
TYR03	42,310	5,282	37,028	88

[Y136]WT: PSNSPPYVCYKK and [F136]MT: PSNSPPFVCYKK which include Tyrosine 136 and Phenylalanine 136, were used as substrate to identify protein tyrosine kinases. % CFC: % change from control.
