# Supplementary material for: Novel roles for LIX1L in promoting cancer cell proliferation through ROS1-mediated LIX1L phosphorylation
Source: Sci Rep. 2015 Aug 27;5:13474. doi: 10.1038/srep13474 (PMC4550850; doi:10.1038/srep13474)
Supplement: Supplementary Information [file srep13474-s1.pdf]

Supplementary Information

Novel roles for LIX1L in promoting cancer cell proliferation through ROS1-mediated LIX1L phosphorylation

Satoki Nakamura, Tomoaki Kahyo, Hong Tao, Kiyoshi Shibata, Nobuya Kurabe, Hidetaka Yamada, Kazuya Shinmura, Kazunori Ohnishi, and Haruhiko Sugimura.

Supplementary Table 1. The identification of the proteins by MALDI-TOF/TOF mass spectrometry

Sample Name.		Protein	gene	MW	Score	Peptide	Coverage	Accession	*Note**
N-p150	1	ATP-dependent RNA helicase A	DHX9 DDX9, LKP, NDH2	140,958	265	23	21	Q08211	*gi|1806048*
N-p100	1	Splicing factor, proline- and glutamine-rich	SFPQ PSF	76,149	157	10	15	P23246	*gi|29881667*
	2	Nucleolin	NCL	76,614	50	3	3	P19338	*gi|189306*
	3	Hornerin	HRNR S100A18	282,390	49	6	4	Q86YZ3	*gi|410171504*
	4	Nucleolar RNA helicase 2	DDX21	87,344	38	4	6	Q9NR30	*gi|1230564*
N-p68	1	Heterogeneous nuclear ribonucleoprotein L	HNRNPL HNRPL	64,133	208	13	33	P14866	*gi|32356*
	2	Heterogeneous nuclear ribonucleoprotein K	HNRNPK HNRPK	50,976	143	7	21	P61978	*gi|460789*
	3	Hornerin	HRNR S100A18	282,390	50	2	1	Q86YZ3	*gi|410171504*

Note : Accession in Mascot Search Results*

The supernatants derived from the nuclear fractions of HEK-293FLG-LIX1L cells treated without RNase were analyzed by MALDI-TOF/TOF mass spectrometry.

Supplementary Table 2. The identification of LIX1L-associated proteins by MALDI-TOF/TOF mass spectrometry.

microRNA List	Database (Algorithm)	Target genes
AC Test	PITA	1,018
	TargetScan	2,226
	microRNA.org	2,798
Z Test	PITA	935
	TargetScan	1,980
	microRNA.org	2,445
Fold change ≥ 2.0	PITA	576
	TargetScan	1,697
	microRNA.org	1,485

The supernatants derived from the nuclear fraction of HEK-293FLG-LIX1L cells treated without RNase were analyzed by MALDI-TOF/TOF mass spectrometry.

Supplementary Figure 1. Representative IHC staining.

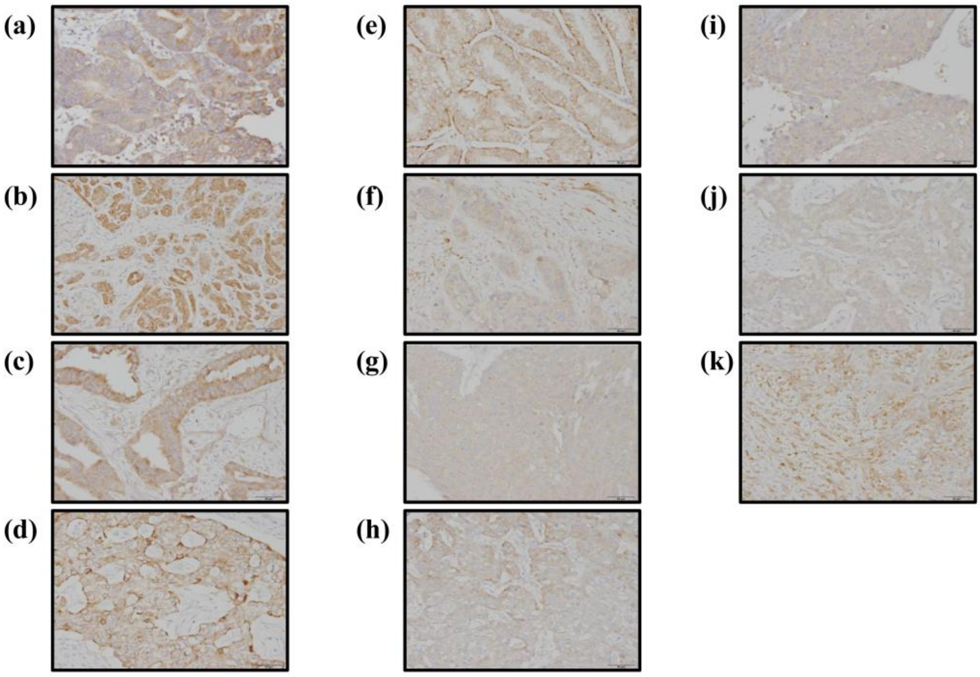

Validation of the IHC staining of LIX1L in cancer tissues**.** Sections were stained for LIX1L using an IHC method. (a) Gastric cancer, (b) Pancreatic cancer, (c) Colon cancer, (d) Ovarian cancer, (e) Kidney cancer, (f) Breast cancer, (g) Lung cancer, (h) Hepatocellular cancer, (i) Esophageal cancer, (j) Prostate cancer, (k) Thyroid cancer. Magnifications: x 400.

Supplementary Figure 2. Representative IHC images of LIX1L in non-cancer tissues.

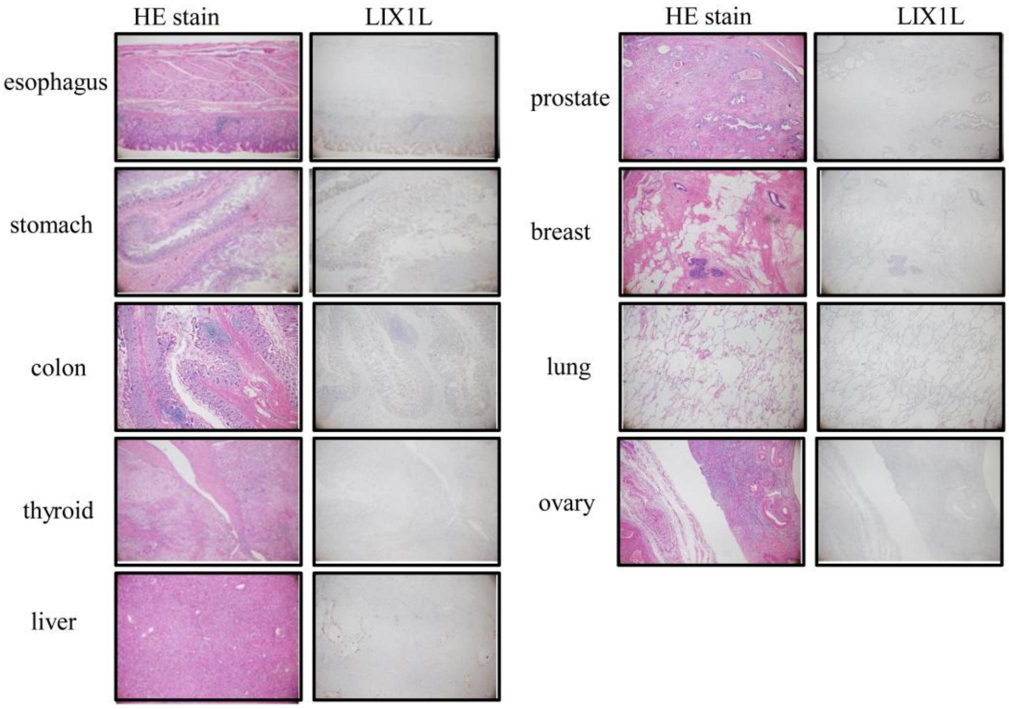

Various non-cancer tissue samples from different patients were evaluated after staining with an anti-LIX1L antibody and HE. The magnification was 400x, and the scale bar represents 50 µm.

Supplementary Figure 3. ***LIX1L*** **knock-down on gastric cancer cell.**

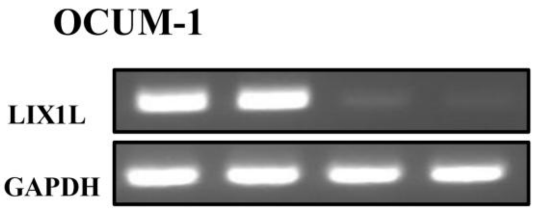

OCUM-1 cells were transfected with scrambled shRNA or *LIX1L* shRNA-#1 or -#2. The RT-PCR results are representative of three independent experiments. The gels were run under the same experimental conditions.

Supplementary Figure 4. The cell proliferation assay using the MTT reagent

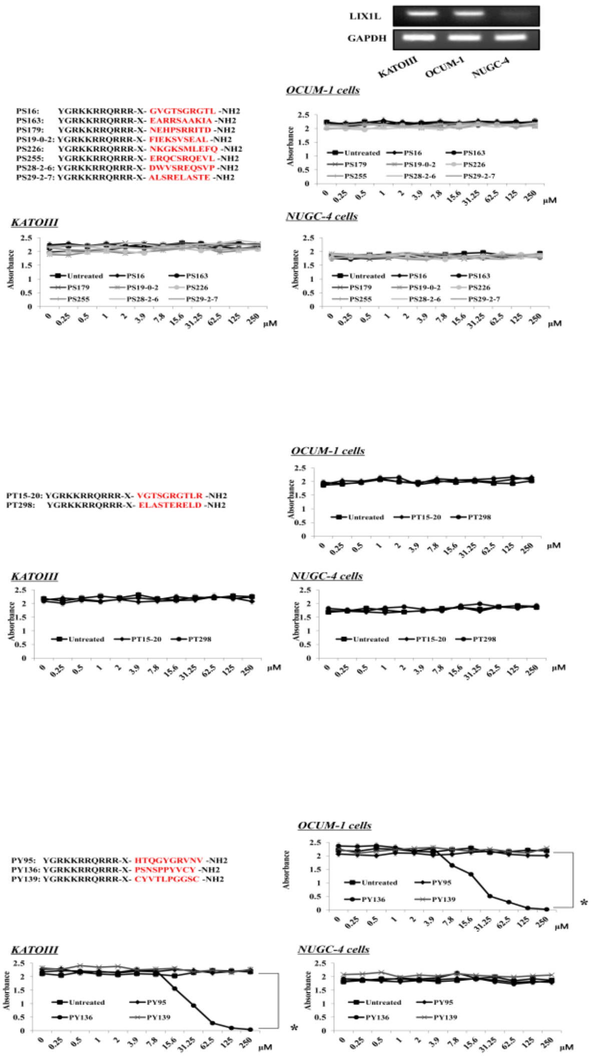

KATOIII, OCUM-1 and NUGC-4 cells were treated with the indicated peptide concentrations for 72 h, and the absorbance was measured. The *LIX1L* mRNA levels in KATO-III, OCUM-1 and NUGC-4 cells were analyzed using quantitative RT-PCR, The RT-PCR results are representative of three independent experiments. $*P < 0.01$ compared with untransfected control cells.

Supplementary Figure 5. Confirmation of FAM-PY136 internalization in LIX1L-deficient NAGC-4 cells.

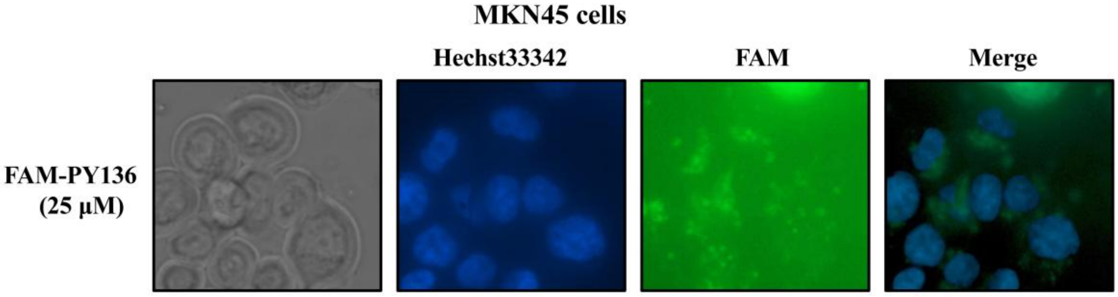

Confirmation of 25 µM FAM-PY136 internalization (green) into non-permeabilized LIX1L-deficient NUGC-4 cells after a three-hour treatment. Nuclei were stained using Hoechst33342 (blue). The cells were viewed using phase-contrast and fluorescence microscopy. Representative images are shown (Magnifications: x 400).

Supplementary Figure 6. The results of the cell proliferation assay in cancer cells treated with random peptides as determined by the MTT assay

METMRAQRLQ PGVGTSGRGT LRALRPGVTG AAAATATPPA GPPPAPPPPA PPPPPLLLSG APGLPLPPGA AGSPAVLREA VEAVVRSFAK HTQGYGRVNV VEALQEFWQM KQSRGADLKN GALVVYEMVP SNSPPYVCYV TLPGGSCFGS FQFCPTKAEA RRSAAKIALM NSVFNEHPSR RITDEFIEKS VSEALASFNG NREEADNPNT GIGAFRFMLE SNKGKSMLEF QELMTVFQLL HWNGSLKAMR ERQCSRQEVL AHYSHRALDD DIRHQMALDW VSREQSVPGA LSRELASTER ELDEARLAGK ELRFHKEKKD ILVLAAGQLG NMHSSNC

	Targeted peptide Sequence
Pcon:	YGRKKKRRQRRR- NH2
Pran1:	YGRKKKRRQRRR-X- LRALRPGVTG-NH2
Pran2:	YGRKKKRRQRRR-X-KHTQGYGRVN-NH2
Pran3:	YGRKKKRRQRRR-X-VVYEMVPSNSP-NH2
PY136:	YGRKKKRRQRRR-X-PSNSPPYVCY-NH2
Pran4:	YGRKKKRRQRRR-X- GSFQFCPTKA -NH2
Pran5:	YGRKKKRRQRRR-X-GNREEADNPN-NH2
Pran6:	YGRKKKRRQRRR-X-DIRHQMALDW -NH2

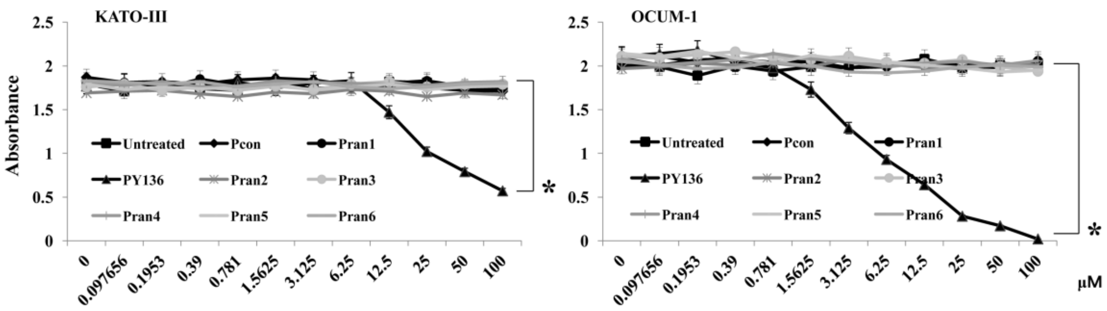

KATO-III and OCUM-1 cells were treated with six random peptides and PY136. Both cell lines exhibited an inhibition of their proliferation by PY136, but not the other peptides. *$P < 0.01$ compared with untransfected control cells.

Supplementary Figure 7. The results of the cell proliferation assay in cancer cells treated with the 10-, 20- and 30-amino acid peptides, as determined by the MTT assay

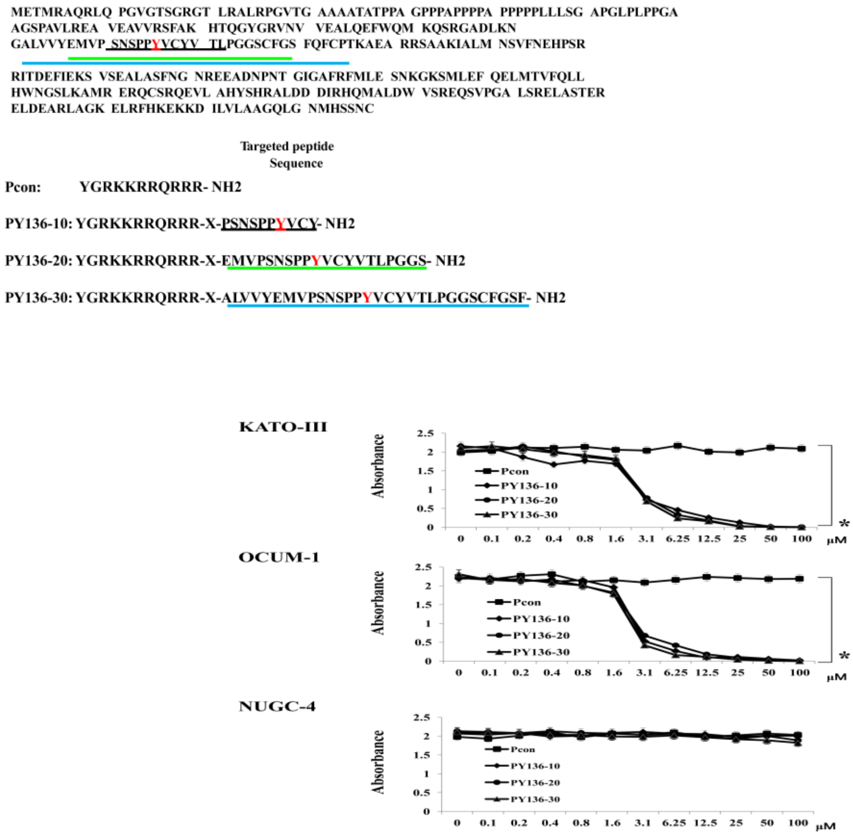

PY136-10 is the same as PY136. PY136-20 and PY136-30 were 20 and 30 amino acids long before and after centering the 136Y, respectively. All three peptides inhibited the proliferation of KATO-III and OCUM-1 cells. In contrast, these peptides did not inhibit the proliferation of NUGC-4 cells. *$P < 0.01$ compared with untransfected control cells.

Supplementary Figure 8. Confirmation of the activity of anti-pT and pS antibodies.

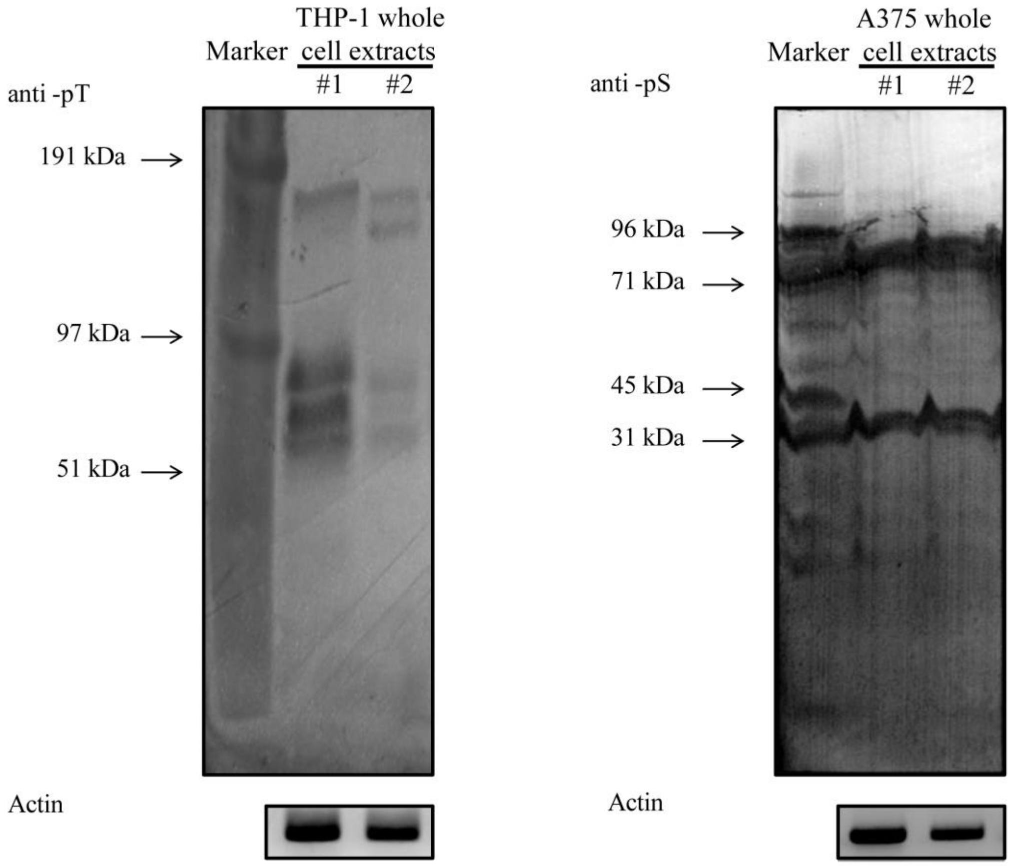

Western blotting of whole cell extract of a human leukemia THP-1 cells and a human melanoma A375 cells with anti-phosphothreonine and anti-phosphoserine antibodies, respectively. Actin level was blotted as an internal standard of cell protein.

Supplementary Figure 9. Confirmation of accurate separation (cytosolic and nuclear fractions).

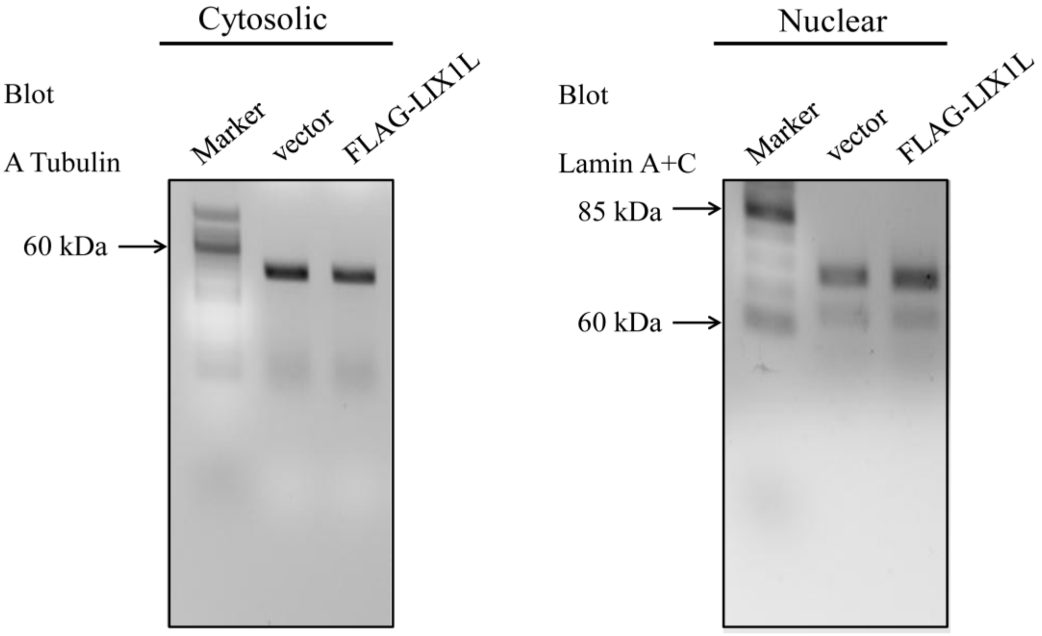

Western blotting by using anti-α Tubulin antibody and anti-Lamin A+C antibody of the cytosolic and nuclear fractions separated from whole cell extract of HEK-293FLG and HEK-293FLG-LIX1L cells, respectively.

Supplementary Figure 10. Determination of cell proliferation by the MTT assay

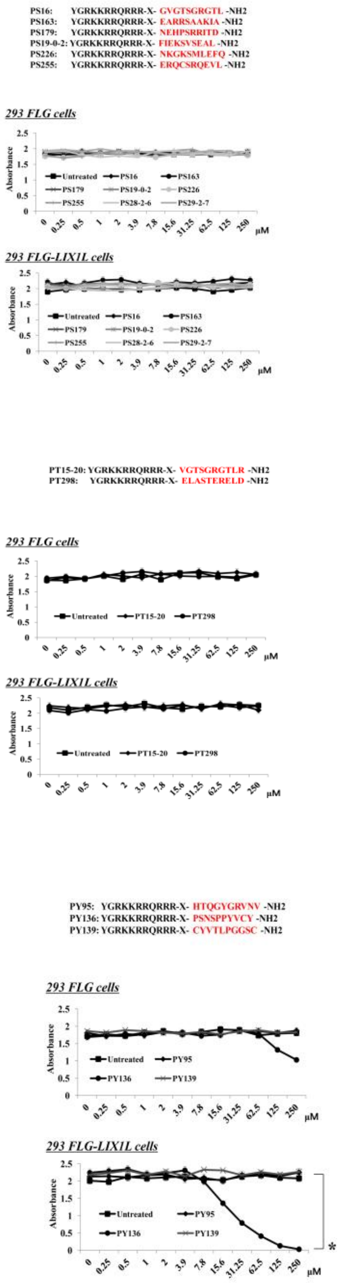

The HEK-293FLG and HEK-293FLG-LIX1L cells were treated with the indicated peptide concentrations for 72 h, and the absorbance was measured. *$P < 0.01$ compared with untransfected control cells.

Supplementary Figure 11. The results of the immunoblot analysis of the nuclear fraction in HEK-293FLG-LIX1L cells with or without treatment with RNase.

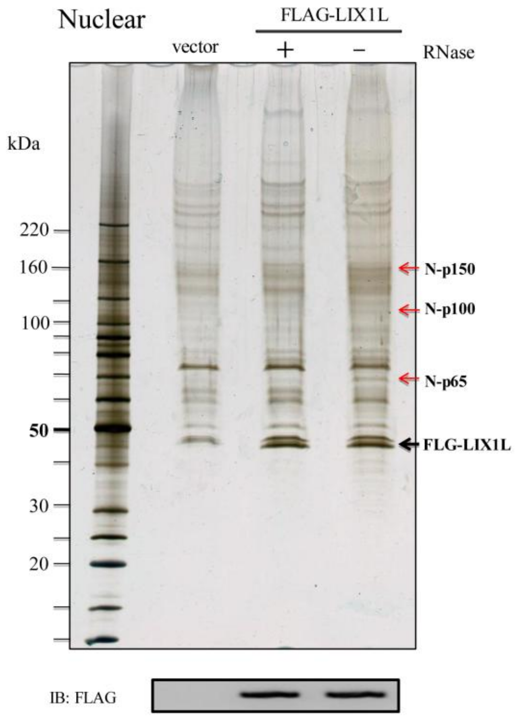

The black arrow indicates the FLAG-LIX1L fusion protein. Red arrows indicate the detected proteins associated with the LIX1L-RNA complex. Proteins of N-p150, N-p100 and N-p65 kDa were detected.

Supplementary Figure 12. LIX1L interacts with the DHX9, nucleolin and hnRNPL proteins in nuclear.

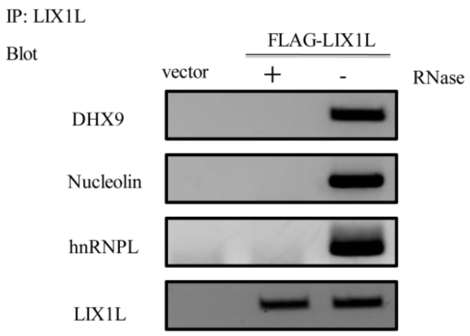

LIX1L interacts with the DHX9, nucleolin and hnRNPL proteins in nucleus of 293 cells, as confirmed using a Western blot analysis. The blots were run under the same experimental conditions.
